# Far-red light effects on plant photosynthesis: from short-term enhancements to long-term effects of artificial solar light

**DOI:** 10.1093/aob/mcae104

**Published:** 2024-07-01

**Authors:** Martina Lazzarin, Killian Dupont, Wim van Ieperen, Leo F M Marcelis, Steven M Driever

**Affiliations:** Horticulture and Product Physiology, Wageningen University, 6700 AA Wageningen, The Netherlands; Horticulture and Product Physiology, Wageningen University, 6700 AA Wageningen, The Netherlands; Horticulture and Product Physiology, Wageningen University, 6700 AA Wageningen, The Netherlands; Horticulture and Product Physiology, Wageningen University, 6700 AA Wageningen, The Netherlands; Centre for Crop Systems Analysis, Wageningen University, 6700 AA Wageningen, The Netherlands

**Keywords:** Artificial solar irradiance, CO_2_ assimilation rates, dynamic photosynthesis, far-red photons, fluctuating lights, morphology, non-photochemical quenching, tomato plants (*Solanum lycopersicum*), photosynthetic active radiation, quantum yield, whole-plant photosynthesis

## Abstract

**Background and Aims:**

Long-term exposure over several days to far-red light (FR) increases leaf expansion, whereas short-term exposure (minutes) might enhance the photosystem II operating efficiency ($\phi_{PSII}$). The interaction between these responses at different time scales and their impact on photosynthesis at the whole-plant level are not well understood. We aimed to assess the effects of FR in an irradiance mimicking the spectrum of sunlight (referred to as artificial solar irradiance), in both the long and short term, on whole-plant CO_2_ assimilation rates and in leaves at different positions in the plant.

**Methods:**

Tomato (*Solanum lycopersicum*) plants were grown under artificial solar irradiance conditions with either a severely reduced or normal fraction of FR [SUN(FR−) vs. SUN]. To elucidate the interplay between the growth light treatment and the short-term reduction of FR, we investigated this interaction at both the whole-plant and leaf levels. At the whole-plant level, CO_2_ assimilation rates were assessed under artificial solar irradiance with a normal fraction and a reduced fraction of FR. At the leaf level, the effects of removal and presence of FR (0FR and 60FR) during transition from high to low light on CO_2_ assimilation rates and chlorophyll fluorescence were evaluated in upper and lower leaves.

**Key Results:**

SUN(FR−) plants had lower leaf area, shorter stems and darker leaves than SUN plants. Although reducing FR during growth did not affect whole-plant photosynthesis under high light intensity, it had a negative impact at low light intensity. Short-term FR removal reduced both plant and leaf CO_2_ assimilation rates, but only at low light intensity and irrespective of the light treatment during growth and the leaf position. Interestingly, the kinetics of $\phi_{PSII}$ from high to low light were accelerated by 60FR, with a larger effect in lower leaves of SUN than in SUN(FR−) plants.

**Conclusions:**

Growing plants with a reduced amount of FR light lowers whole-plant CO_2_ assimilation rates at low light intensity through reduced leaf area, despite maintaining similar leaf-level CO_2_ assimilation to leaves grown with a normal amount of FR. The short-term removal of FR brings about significant but marginal reductions in photosynthetic efficiency at the leaf level, regardless of the long-term growth light treatment.

## INTRODUCTION

Far-red light (FR, 700–750 nm) has traditionally been excluded from the definition of photosynthetically active radiation (PAR) because the spectral quantum yield curve of photosynthesis (CO_2_ assimilation rates per unit absorbed energy per wavelength) declines above 700 nm (McCree, 1971). Recently, it has been suggested to expand what is considered PAR (Zhen and Bugbee, 2020*a*, *b*). Zhen and Bugbee (2020*b*) indicated that FR photons are as efficient as PAR in driving canopy photosynthesis on an absorbed basis when substituted in a white light spectrum. In the short-term, FR increases net leaf CO_2_ assimilation rates. The extent of this effect is influenced not only by the background light spectrum during measurement, but also by the light spectrum and intensity under which the leaves have developed. Lettuce (*Lactuca sativa*) leaves grown under red/blue LED lights showed a larger increase in photosystem II (PSII) operating efficiency ($\phi_{PSII}$) when FR was added to the measuring light, compared with sunlight-grown lettuce (Zhen and van Iersel, 2017). In tomatoes (*Solanum lycopersicum*) grown in the greenhouse, under sunlight with supplemental red/blue LED lights with varying FR levels, this enhancement effect was negligible (Kalaitzoglou *et al.*, 2019). In contrast, when FR was removed from the sunlight irradiance during measurement in sunflower (*Helianthus annuus*) and corn leaves (*Zea mays*), leaf net CO_2_ assimilation rates decreased, and the size of this effect decreased with increasing light intensity (Zhen *et al.*, 2022). In these studies, plants were grown in the presence of FR. It is uncertain whether growing plants without or with very little FR light in the solar irradiance (a condition not occurring in nature) would enhance the short-term effect of FR on leaf photosynthesis.

In the long term, FR accelerates leaf appearance rate, leaf expansion and stem elongation (Hogewoning *et al.*, 2010; Demotes-Mainard *et al.*, 2016). These morphological responses enhance light interception by the plant (Kalaitzoglou *et al.*, 2019). Concomitantly, the plant morphology also affects the vertical gradient in light intensity within the plant. Lower leaves acclimate to low light intensity and, as a result, have lower photosynthetic capacity compared with upper leaves (Trouwborst *et al.*, 2011). Additionally, non-photochemical quenching (NPQ) capacity is higher in lower leaves compared with upper leaves (Foo *et al.*, 2020). Further complexities arise because the intensity of FR decreases less strongly compared with red light, resulting in a decreased red-to-FR ratio (R/FR) towards the lower leaves compared with the upper leaves (Zhang *et al.*, 2020). Moreover, light intensity plays a role, because leaf photosynthetic efficiency is lowered when growing tomato plants with FR light at low light intensity, but not when grown at high light intensity (Wassenaar *et al.*, 2022). Although long-term exposure to FR reduces photosynthetic capacity, it is noteworthy that several observations were made without FR in actinic light (Ji *et al.*, 2019). Therefore, the long-term effect of FR on leaf photosynthesis remains unclear.

At the leaf level, FR decreases leaf absorptance in the PAR range, reduces leaf thickness and reduces chlorophyll/carotenoid content (Bou-Torrent *et al.*, 2015; Kalaitzoglou *et al.*, 2019; Zhen *et al.*, 2019; Hu *et al.*, 2021). At the chloroplast level, FR temporarily enhances photosynthesis through the known Emerson enhancement effect (Emerson *et al.*, 1957). Given that the absorbance properties differ for photosystem I (PSI) and PSII, the balance of excitation between photosystems is wavelength dependent (Evans, 1986; Chow *et al.*, 1990; Hogewoning *et al.*, 2012). FR rebalances the excitation pressure between photosystems by exciting PSI preferably, thereby increasing the quantum yield.

The short-term effect of FR becomes even more complex when considering leaves in different positions on the plants. This complexity originates from the fact that leaves tune their photosystem stoichiometry to the growth light spectrum to maximize the light-use efficiency for a given shade or full sun spectrum (Chow *et al.*, 1990). For example, shade-adapted leaves, grown under a light spectrum with a higher fraction of FR, have a higher light-use efficiency when illuminated with FR light than sun-adapted leaves (Taylor *et al.*, 2019). Conversely, sun-adapted leaves are more efficient in driving photosynthesis with a low fraction of FR or even without FR in the given light spectrum (Taylor *et al.*, 2019). Due to limited data, it remains unclear whether the short-term removal of FR would decrease leaf photosynthetic rates more strongly in shaded leaves than in the sun-adapted leaves or how this interacts with changes in light intensity. This requires disentangling the long-term effects of FR (morphological responses important for light interception) from the short-term responses [e.g. increase in PSII operating efficiency ($\phi_{PSII}$) owing to improved excitation balance] at different leaf positions. Given the intricate interplay between variations in light intensity and the amount of FR among different leaf positions, the scaling of responses to FR from individual leaves to whole-plant photosynthesis is extremely complex.

The aim of this study was to quantify the impact of reducing the amount of FR light in the solar light irradiance, in both the long and short term, on photosynthesis of whole plants and of leaves at different positions. First, we hypothesized that long-term removal of FR during growth would result in plants with reduced leaf area and stem height, but would be compensated at the whole-plant level by higher leaf photosynthetic rates in comparison to plants grown with normal FR light. Second, we hypothesized that the short-term removal of FR would decrease leaf photosynthetic rates by a reduction in $\phi_{PSII}$ and that the size of this effect would increase vertically from top to bottom with leaf position.

Tomato plants (*Solanum lycopersicum*) were grown in climate chambers for ~1 month with either a severely reduced fraction of FR [SUN(FR−)] or a normal fraction of FR (normal for direct solar irradiance; SUN) in artificial solar irradiance. This irradiance was designed to mimic solar irradiance and thereby leads to a wavelength distribution that excites both photosystem PSI (>680 nm) and PSII (<680 nm) (Evans, 1986, 1987), in contrast to a spectrum containing mainly PSII-exciting light (~80 % of FR photons in the 701–750 nm region were removed). Whole-plant CO_2_ assimilation rates were assessed for plants of both growth light treatments, at both high and low light intensity. Leaf CO_2_ assimilation rates were evaluated for leaves in the upper and lower positions of the plants, with and without FR, and at high and low light intensity. Simultaneously, chlorophyll fluorescence was used to evaluate transient changes during transition from high to low light.

## MATERIALS AND METHODS

### Plant material and growth conditions during germination

Tomato (*Solanum lycopersicum* cv. Moneymaker) seeds were sown in stone wool plugs (Grodan, Roermond, The Netherlands) presoaked in 50 % strength tomato nutrient solution, diluted with demi water (electrical conductivity 1 dS m^−1^, pH 5.5). Full-strength nutrient solution contained: 1.2 mM NH_4_^+^, 7.2 mM K^+^, 4.0 mM Ca^2+^, 1.8 mM Mg^2+^, 12.4 mM NO_3_^−^, 3.3 mM SO_4_^2−^, 1.0 mM PO_4_^2−^, 35 μM Fe^3+^, 8.0 μM Mn^2+^, 5.0 μM Zn^2+^, 20 μM B, 0.5 μM Cu^2+^ and 0.5 μM MoO_4_^2−^. The stone wool plugs with seeds were placed in polystyrene trays with a 1–2 cm layer of nutrient solution, covered with vermiculite and stored at 4 °C in darkness for 48 h. Plugs with seeds were then transferred to a growth chamber to germinate for 11 days at 22 °C/18 °C (day/night), 70 % relative humidity, a constant intensity of 150 µmol m^−2^ s^−1^ photosynthetic photon flux density (PPFD) and 16-h photoperiod (8.64 mol m^−2^ day^−1^ daily light integral), supplied by fluorescent lamps (Philips MASTER TL-D Super 80 58W/840, Signify, The Netherlands).

### Light spectrum treatments and growth conditions

**Fig. 1.**
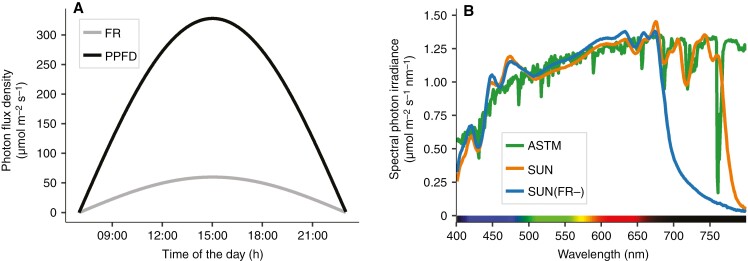
(A) Diurnal parabolic growth light regime of the photosynthetic photon flux (PPFD, 400–700 nm) and the far-red photon flux density (FR, 700–750 nm). (B) Spectral photon irradiance with either a normal fraction of FR (SUN) and with severely reduced FR [SUN(FR−)] in the artificial solar irradiance compared with the American Society for Testing and Materials (ASTM) spectrum (ASTM G-173, at PAR wavelengths, http://www.astm.org/g0173-03.html) measured at 325 µmol m^−2^ s^−1^ (PPFD).

After 11 days, uniform tomato seedlings (with fully opened cotyledons and the first true leaf not yet visible) were selected, transferred to rockwool blocks (7.5 cm × 7.5 cm × 6.5 cm; Grodan, Roermond, The Netherlands) and moved to custom-made climate cabinets (SOLINATOR). Two growth light treatments were given with similar PPFD but contrasting supplementation of FR: artificial solar irradiance with a normal fraction of FR (SUN) and with a severely reduced FR [SUN(FR−); Supplementary Data Table S1]. Both growth light treatments had a parabolic, diurnally variable irradiance intensity profile of up to a maximum of 325 µmol m^−2^ s^−1^ (PPFD), a 16-h photoperiod (Fig. 1A) and a daily light integral of ~13 mol m^−2^ day^−1^. The irradiance intensity during growth was continuously monitored and maintained at the level of the top leaf of the plants using a PAR quantum sensor (LI-190R).

In the SOLINATOR cabinets, growth light was supplied by artificial solar lamps (artificial sunlight research modules generation 1 and 2, Specialty Lighting Holland BV, Breda, The Netherlands) with a PAR spectrum (400–700 nm) similar to the spectral distribution of standardized direct solar irradiance (ASTM G-173, at PAR wavelengths, http://www.astm.org/g0173-03.html). Realized spectra in both growth light treatments (Fig. 1B) were measured with a spectroradiometer (FLAME-T + UV-VIS optics, Ocean Optics, Duiven, the Netherlands). FR levels were quantified using the phytochrome stationary state (PSS), based on absorbance spectra of phytochrome in activated and inactivated state, taking into account a much wider wavelength range than R/FR ratios (Sager *et al.*, 1988). A PSS value of 0.72 was realized for the SUN (equivalent to the PSS of direct solar irradiance) and 0.83 for the SUN(FR−) growth light treatment (Supplementary Data Table S1).

Plants were grown for 22–25 days in the SOLINATOR at 22 °C/18 °C (day/night) and 65 % relative humidity, and they were irrigated with 50 % strength nutrient solution three times a week, until the fourth leaf was fully developed. The day before measurements, plants were irrigated to avoid any stomatal limitation the following day. Plants used for measurements of gas exchange and chlorophyll fluorescence were transferred to the laboratory in the middle of the light period, coinciding with the highest growth light intensity (Fig. 1A).

### Whole-plant gas-exchange measurements

**Fig. 2.**
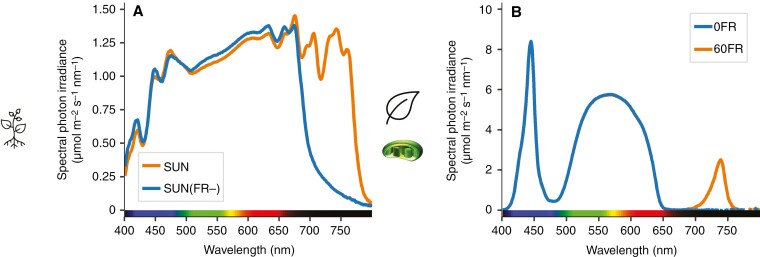
Light spectra used for the gas-exchange and chlorophyll-fluorescence measurements. (A) The artificial solar irradiance {with normal FR (SUN) or reduced FR [SUN(FR−)]} was used for the assessment of whole-plant CO_2_ assimilation rates during transition from high to low light intensity. (B) White actinic light with either no FR (blue line, 0FR) or with 60 µmol m^−2^ s^−1^ FR added (blue and orange line, 60FR) was used for the combined assessment of leaf CO_2_ assimilation rates and chlorophyll fluorescence from high to low light intensity. In the example spectra shown, the artificial solar irradiance intensitywas 325 µmol m^−2^ s^−1^ (PPFD) (A), and white light was 850 µmol m^−2^ s^−1^ (PPFD) (B).

Whole-plant CO_2_ assimilation rates were assessed on 22- to 25-day-old plants using a light source with a spectrum similar to that during growth (Fig. 2A; Supplementary Data Table S2) and a custom-made gas-exchange system. The plant was mounted inside a clear plexiglass chamber [29 cm in diameter, 18 or 27 cm high, for SUN(FR−) or SUN plants, respectively] lined with a fluorinated ethylene propylene foil (Holscot Europe, Breda, The Netherlands), placed inside a cabinet (63 cm × 63 cm) with white reflective walls. Air mixing was ensured by three fans (SanAce 40 W, type 9WL0424P3J001, Sanyo Denki, Philippines) at the bottom of the chamber. The minimum chamber air temperature was maintained at 25 °C by heating cables. Synthetic air containing 21 % O_2_ was supplied using mass flow controllers (EL-Flow, Bronkhorst, Buurlo, The Netherlands), at a flow rate of 10 L air min^−1^ (7.436 mmol s^−1^), and CO_2_ and H_2_O of incoming and outgoing air were measured using an infra-red gas analyser (LI-7000, LI-COR Biosciences, Lincoln, NE, USA). The CO_2_ concentration was maintained at ~375 ppm and relative humidity at ~75 % in the plant chamber (achieved conditions are given in Supplementary Data Table S3). Vertical and horizontal PPFD distributions in the empty plant chamber are given in Supplementary Data Figs S1 and S2.

The whole-plant net photosynthetic CO_2_ assimilation rate ($P_{n,plant}$, in micromoles of CO_2_ per square metre per second) was calculated from whole-plant CO_2_ and H_2_O gas exchange according to Field *et al.* (1989):


$$\begin{array}{l} \;P_{n,plant}\;=\;\frac{F}{S}\;\times \left(CO_{2\;reference}-CO_{2\;sample}\times \frac{\left(1000-H_{2}O{}_{reference}\right)}{\left(1000-H_{2}O{}_{sample}\right)}\right) \end{array}$$ (1)

where *F* (in moles per second) is the molar flow rate of air entering the chamber, *S* (in square metres) is the total leaf area of the plant, $CO_{2\;reference}$ and $CO_{2\;sample}$ are the mole fraction of CO_2_ (in micromoles per mole of air) in the reference and sample infra-red gas analyser, respectively. The $H_{2}O{\;}_{reference}$ and $H_{2}O{\;}_{sample}$ are the mole fraction of water vapour (in millimoles of H_2_O per mole of air) in the reference and sample IRGA, respectively.

Prior to measurements, SUN-grown plants were light acclimated at a light intensity of 660 ± 12 µmol m^−2^ s^−1^ PPFD (mean ± s.d.) with supplemental FR light (124 µmol m^−2^ s^−1^, PSS = 0.72, R:FR = 1.1). SUN(FR−)-grown plants were acclimated at the same PPFD, but with a reduced amount of supplemental FR (24 µmol m^−2^ s^−1^, PSS = 0.83, R:FR = 6.8). The amount of FR supplemented was chosen to resemble the same PSS as in the growth light treatments. Each plant was acclimated for ~60 min until a constant $P_{n,plant}\;$ was achieved. Plant CO_2_ assimilation rates ($P_{n,plant}\;$) were then measured every minute (averaging time 10 s) for 20 min at high PPFD (660 ± 12 μmol m^−2^ s^−1^) followed by 20 min of low PPFD (100 ± 2.6 μmol m^−2^ s^−1^). This was repeated, once in the presence of FR (124 and 19 μmol m^−2^ s^−1^ of FR, respectively, PSS = 0.72, FR+) and once with severely reduced FR (24 and 4 μmol m^−2^ s^−1^ of FR, respectively, PSS = 0.83, FR−).

Measurements starting with or without FR were alternated between different plants, and high and low light was alternated. A total of six SUN- and six SUN(FR−)-grown plants were measured. The FR level during measurement was quantified using a spectroradiometer (as specified above) placed at the level of the top leaf. After whole-plant gas-exchange measurements, leaves were removed from the plant, and the area of each leaf was measured by scanning the leaf on graph paper. The scanned image was then analysed using ImageJ (Schneider *et al.*, 2012), excluding the petioles, petiolules and the rachis. Stem height was measured from the apex to the hypocotyl.

### Combined leaf gas-exchange and chlorophyll-fluorescence measurements

The combined measurements of gas exchange and chlorophyll fluorescence at the leaf level were done on the second leaflet of the second (lower) and fourth (upper) appearing leaves of 25-day-old plants. Light spectra differing from growth light were used during measurements: white actinic light alone (0FR) or white supplemented with FR (60FR) (Fig. 2B; Supplementary Data Table S4). Chlorophyll fluorescence was measured using a FluorCam closed chlorophyll fluorescence imaging system (FC 800-C, Photon Systems Instruments, Brno, Czech Republic). Gas exchange was measured using a LI-6400XT gas-exchange system (Li-Cor Biosciences, Lincoln, NE, USA) with a standard 2 cm × 3 cm clear-topped cuvette modified with glass with an anti-reflective coating (UQG OPTICS, Cambridge, UK). The leaf was clamped in the clear-topped cuvette centred underneath the FluorCam light source to measure gas exchange and chlorophyll fluorescence simultaneously. During measurements, block temperature was controlled at 22 °C, air flow at 300 µmol s^−1^ and CO₂ reference at 400 µmol mol^−1^ (resulting environmental conditions are given in Supplementary Data Table S5).

The leaf was first dark acclimated for ≥20 min [to reach a maximum quantum efficiency of PSII photochemistry ($\;F_{V}/F_{m}\;$) ≥ 0.80 and measure dark respiration rate ($R_{d}$)]. Subsequently, the leaves of SUN-grown plants were light acclimated with 60 µmol m^−2^ s^−1^ FR (60FR) light added to 850 µmol m^−2^ s^−1^ PPFD for 30–60 min until net leaf CO_2_ assimilation rate ($P_{n,\;leaf}$) and stomatal conductance ($g_{s}\;$) were stable. The SUN(FR−)-grown plants were acclimated without FR (0FR) light at 850 µmol m^−2^ s^−1^ PPFD (Fig. 2B; Supplementary Data Table S4). After acclimation, in the 0FR treatment, the leaf was exposed to 10 min of high light (850 ± 53 µmol m^−2^ s^−1^ PPFD) and 15 min of low light (60 ± 7.8 µmol m^−2^ s^−1^ PPFD), during which gas exchange and chlorophyll fluorescence were measured.

Gas exchange was recorded every minute (averaging time 10 s). The quantum yield of CO_2_ assimilation ($\phi_{CO_{2\;\;\;}}$) was determined using the $P_{n,\;leaf}$ 12 min after the onset of low light; the quantum yield of CO_2_ assimilation, $\Phi_{CO_{2}}$, is calculated as:


$$\begin{array}{l} \Phi_{CO_{2}}=\frac{P_{n,leaf}+R_{d}}{I_{abs\;\;\;}} \end{array}$$ (2)

where $P_{n,leaf}$ is the net leaf CO_2_ assimilation rate (in micromoles of CO_2_ per square metre per second), $R_{d}$ is the dark respiration rate, and $I_{abs\;\;\;}$ is the absorbed light intensity.

For chlorophyll fluorescence, the measuring beam was set at 0.25 µmol m^−2^ s^−1^ and saturating pulses (800 ms, light intensity ~3600 µmol m^−2^ s^−1^) were given every 60 s during high light. Upon changing from high to low light intensity, saturating pulses were given in intervals of 10, 30, 30, 60, 60, 120, 120, 120, 120, 120 and 120 s. To avoid interference of FR with the measured chlorophyll fluorescence signal, FR was temporarily turned off when determining steady-state fluorescence emission ($F^{\prime }\;$) and maximal fluorescen emission of a light adapted leaf ($\;{F^{\prime }}_{m}$.)

Chlorophyll fluorescence parameters were calculated according to Baker (2008). The maximum quantum efficiency of PSII photochemistry was calculated as:


$$\begin{array}{l} F_{v}/F_{m}=\left(F_{m}-F_{o}\right)/F_{m}\; \end{array}$$ (3)

where minimum fluorescence (*F*_o_), measured in the dark-adapted state, was determined after the leaf had been subjected to ≥20 min of darkness in the leaf chamber and immediately before the measurement of the maximal fluorescence in the dark-adapted state (*F*_m_). For the light-adapted state, the operating efficiency of PSII, $\phi_{PSII}$, was calculated as:


$$\begin{array}{l} \phi_{PSII}=\left({F^{\prime }}_{m}-F^{\prime }\right)/F^{\prime }\; \end{array}$$ (4)

where ${F^{\prime }}_{m}$ is the maximal fluorescence measured in the light-adapted leaf and *F*ʹ the fluorescence emission from the light-adapted leaf.

The non-photochemical quenching, NPQ, was calculated as:


$$\begin{array}{l} NPQ=\left(F_{m}/{F^{\prime }}_{m}\;\right)-1 \end{array}$$ (5)

The PSII efficiency factor (fraction of open PSII centres), ${F^{\prime }}_{q}/{F^{\prime }}_{v}\;$, was calculated as:


$$\begin{array}{l} \;\frac{{F^{\prime }}_{q}}{{F^{\prime }}_{v}}=\frac{F^{\prime }-\;F^{\prime }}{{F^{\prime }}_{m}-{F^{\prime }}_{0}} \end{array}$$ (6)

Where ${F^{\prime }}_{0}$ is the minimal fluorescence in the light-adapted leaf and was calculated after Oxborough and Baker (1997):


$$\begin{array}{l} F^{\prime }=F_{0}/\left(F_{v}/F_{m}+F_{0}/{F^{\prime }}_{m}\;\;\right)\; \end{array}$$ (7)

The measurement procedure (dark acclimation, light acclimation, 10 min of high light and 15 min of low light) was repeated for each leaf, once in the presence (60FR) and once in the absence of FR (0FR). At high light, the corresponding PSS and R:FR were 0.757 and 0.014, respectively. At low light, the PSS and R:FR were 0.346 and 0.004, respectively (Fig. 2B; Supplementary Data Table S4). The order of 0FR and 60FR was alternated between replications. FR at the leaf level was quantified using a spectroradiometer (as described above). Seven SUN- and six SUN(FR−)-grown plants were measured. Leaf area was determined by taking a picture of the measured leaflet on graph paper and analysing it using ImageJ.

### Leaf absorptance

For the measurement of reflectance and transmittance, three leaf discs (0.79 mm^2^) were punched from each upper and lower leaf of six SUN- and six SUN(FR−)-grown plants. A halogen white and blue LED light source was used, connected by an optical fibre to two separate integrating spheres (50-mm-diameter reflectance and transmittance spheres, Avantes, Appeldoorn, The Netherlands). One sphere was set up for leaf reflectance and one for leaf transmittance measurements. Each sphere was connected to a spectrometer (Ocean Optics USB4000) by an optical fibre and controlled by custom software (Labview, National Instruments Corporation, Austin, TX, USA). Leaf light absorptance (*α*) was then calculated per nanometre from reflectance and transmittance measurements for wavelengths between 400 and 800 nm. The absorbed light was calculated by multiplying the intensity by *α* in 1 nm steps, as previously described (Hogewoning *et al.*, 2010).

### Statistical set-up and analysis

The statistical set-up was a completely randomized design, treating individual plants as repetitions. Data were analysed and visualized with the Tidyverse packages (Wickham *et al.*, 2019) using R (v.4.2.2) and RStudio (2023.06.0). A mixed linear model was fitted with REML using the lme4 package (Bates *et al.*, 2015) to examine the main effects and interaction between two factors: the growth treatment (growing plants with a normal and severely reduced effect of FR light) and the short-term presence or absence of FR light on leaf or whole-plant photosynthetic rates. The measurements with and without FR light were made on the same leaf or plant; therefore, the repeated measurement was included as random intercepts. For the chlorophyll fluorescence parameters from high to low light, the model was fitted at each time point. The model assumptions were assessed by visually inspecting residual vs. fitted plots and the *Q–Q* plots for the residuals and the random effect. *P*-values were obtained with *F*-tests using type II sums of squares with the lmerTest package (Kuznetsova *et al.*, 2017) using the Satterthwaite approximation for unbalanced data. *P*-values for contrasts were Bonferroni-corrected *t*-tests obtained with the emmeans package (Lenth, 2016).

## RESULTS

### Reduced FR during growth increased leaf absorptance but decreased stem length and leaf area

**Fig. 3.**
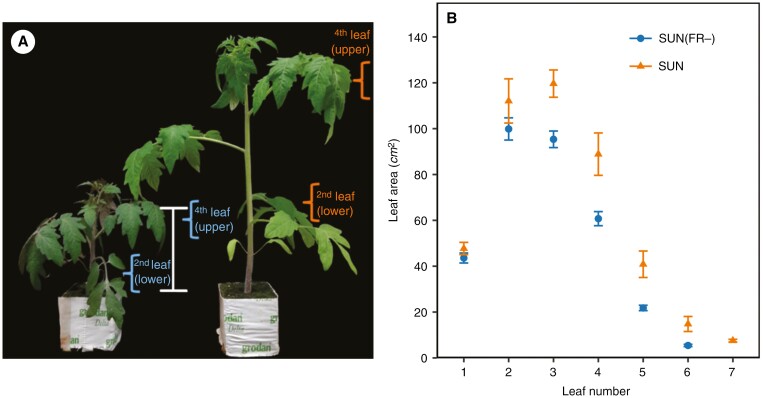
One-month-old SUN(FR−)-grown plants (left) and SUN-grown plants (right). The white bar represents 10 cm. (A) Leaf area of all individual leaves counting from the lower to the upper leaves in SUN(FR−)-grown plants and in SUN-grown plants. (B) Leaf number 2 (older) and leaf number 4 (younger) were chosen for the gas-exchange and chlorophyll-fluorescence measurements. Data are averages ± s.e.m. (*n* = 6).

Plants grown under solar irradiance with normal FR (SUN) were more elongated (30 ± 0.99 cm) than plants grown under solar irradiance with severely reduced FR [SUN(FR−); 13 ± 0.63 cm; Fig. 3A]. SUN-grown plants had a higher total leaf area compared with SUN(FR−) (427 ± 24 vs. 327 ± 11 cm^2^, *P* < 0.001). Additionally, SUN-grown plants had a higher number of leaves per plant and a larger leaf area per leaf than SUN(FR−)-grown plants (Fig. 3B). The leaf absorptance in the PAR (400–700 nm) region was significantly lower in SUN leaves compared with SUN(FR−) leaves (*P* < 0.001; Supplementary Data Fig. S3). In SUN(FR−)-grown plants, the absorbed irradiance between 400 and 700 nm increased by 3.4 and 4.8 % in upper leaves (fourth layer) and lower leaves (second layer) compared with SUN-grown plants (Supplementary Data Table S6). The leaf absorptance in the FR (701–800 nm) was increased by 16.6 and 25.9 % in upper and lower leaves, respectively, of SUN- compared with SUN(FR−)-grown plants (Supplementary Data Table S6).

### The effect of short- and long-term reduction of FR in solar irradiance on whole-plant CO_2_ assimilation rate was light intensity dependent

**Fig. 4.**
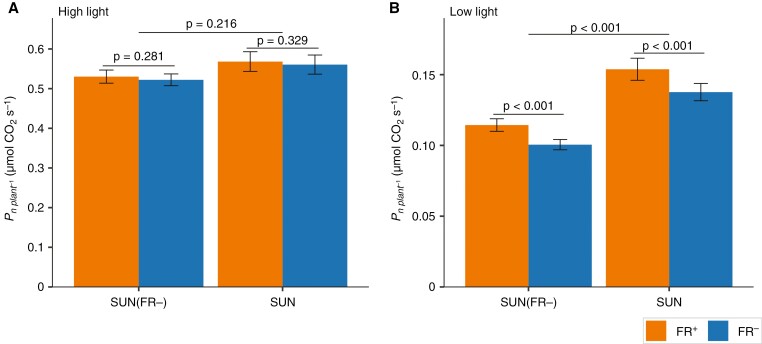
Effects of FR in solar irradiance on the net CO_2_ assimilation rate of young tomato plants ($P_{n,per\;plant}$) grown under artificial solar irradiance with severely reduced FR [SUN(FR−)] or a normal amount of FR (SUN). The values of$P_{n,per\;plant}$ (at steady state) of light-acclimated plants were measured after 20 min exposure to high PPFD (660 µmol m^−2^ s^−1^; A) and after 20 min exposure to low PPFD (100 µmol m^−2^ s^−1^; B) with a normal (FR+, orange) or reduced FR (FR−, blue), as specified in Supplementary Data Table S2. Data are averages ± s.e.m. calculated from raw data (*n* = 6). The *P*-value for the growth treatment was obtained from the mixed model, and a Bonferroni-corrected pairwise comparison was conducted between FR^+^ and FR−.

The SUN(FR−)-grown plants exposed to high light intensity (Fig. 4A) had similar whole-plant CO_2_ assimilation rates ($P_{n,\;per\;plant}$) to SUN-grown plants (Fig. 4A). Whether FR during measurement was reduced (FR−) or not (FR+) had no effect on $P_{n,\;per\;plant}$ at high light (Fig. 4A).

At low light intensity, SUN(FR−)-grown plants had significantly lower whole-plant CO_2_ assimilation rates than SUN-grown plants (Fig. 4B). Reducing FR light (FR−) during measurements compared with a normal amount of FR in solar irradiance significantly reduced whole-plant CO_2_ assimilation rate at this low light intensity, by 12 % for SUN plants and 14 % for SUN(FR−) plants (*P*-value for interaction = 0.45; Fig. 4B).

### Reducing the amount of FR in the solar light spectrum increases whole-plant photosynthesis at high light intensity when normalized by total leaf area

**Fig. 5.**
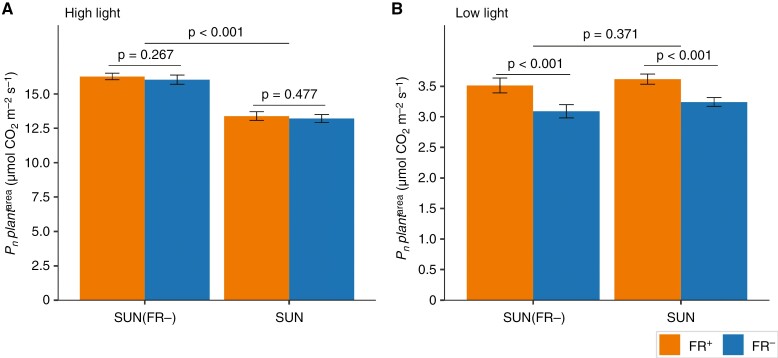
Effects of FR on net whole-plant CO_2_ assimilation rate per total unit leaf area (*P*_n,plant_^area^) in young tomato plants grown under artificial solar irradiance with severely reduced FR [SUN(FR−)] or a normal amount of FR (SUN). The values of *P*_n,plant_^area^ (at steady state) of light-acclimated plants were measured after 20 min exposure to high PPFD (660 µmol m^−2^ s^−1^; A) and after 20 min exposure to low PPFD (100 µmol m^−2^ s^−1^; B) with a reduced FR (FR−, blue), as specified in Supplementary Data Table S2. Data represent the mean ± s.e.m. calculated from raw data [*n* = 6 for SUN(FR−)- and SUN-grown plants]. The *P*-value for the growth treatment was obtained from the mixed model, and a Bonferroni-corrected pairwise comparison was conducted between FR+ and FR−.

To account for the difference in total leaf area between SUN plants and SUN(FR−)-grown plants, whole-plant CO_2_ assimilation rates (Fig. 4) were normalized by the total leaf area. At high light intensity, SUN(FR−)-grown plants had higher whole-plant CO_2_ assimilation per unit area (*P*_n,plant_^area^) than SUN-grown plants (Fig. 5A). When subjected to low light intensity, SUN-grown plants had a similar *P*_n,plant_^area^ to SUN(FR−)-grown plants (Fig. 5B). The reduction of FR during measurements (FR−) did not decrease *P*_n,plant_^area^ at high light intensity in both SUN- and SUN(FR−)-grown plants (Fig. 5A), whereas significant reductions were observed at low light intensity, by 14 and 12 % for SUN and SUN(FR−) plants, respectively (*P*-value of interaction = 0.49; Fig. 5B).

### *The effect of removal of FR on leaf CO*_*2*_  *assimilation rate in upper and lower leaves in SUN- and SUN(FR*−*)-grown plants*

To uncover the underlying mechanisms contributing to the differences observed in whole-plant CO_2_ assimilation rates between SUN- and SUN(FR−)-grown plants, we investigated the long-term effect of reducing FR light on CO_2_ assimilation rates of leaves located at the top (upper) and bottom of the plant (lower) under both high and low light intensity. Additionally, to disentangle the long-term effect of FR light from the short-term response, we measured with and without FR (60FR and 0FR, respectively) in SUN- and SUN(FR−)-grown plants.

**Fig. 6.**
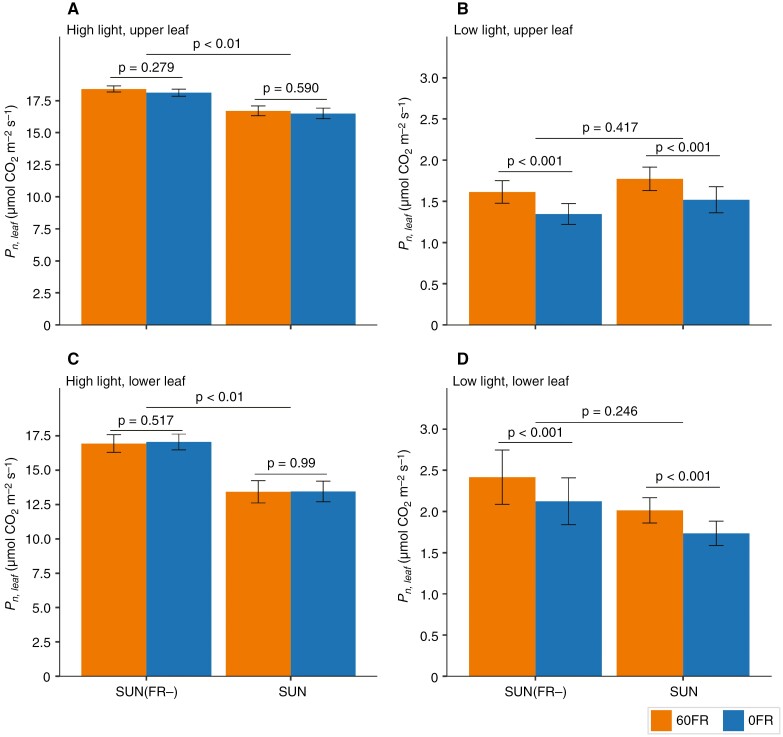
Effects of reducing FR on net CO_2_ assimilation rates ($P_{n,leaf}$, in micromoles of CO_2_ per square metre per second) of upper and lower leaves of young tomato plants grown under artificial solar irradiance with either a normal amount of FR (SUN) or with a severely reduced amount of FR [SUN(FR−)]. The steady-state $P_{n,leaf}$ was assessed after 10 min of high PPFD at 850 µmol m^−2^ s^−1^ and 15 min of low PPFD at 60 µmol m^−2^ s^−1^ in the presence of supplementation of 60 µmol m^−2^ s^−1^ FR (60FR) or absence of FR (0FR) during measurements, as specified in Supplementary Data Table S4. The different colours indicate the presence or absence of FR light (60FR and 0FR) during the measurements. (A) Upper leaf (fourth leaf) of SUN(FR−)- and SUN-grown plants was measured at high light intensity. (B) Upper leaf (fourth leaf) of SUN(FR−)- and SUN-grown plants was measured at low light intensity. (C) Lower leaf (second leaf) of SUN(FR−)- and SUN-grown plants was measured at high light intensity. (D) Lower leaf (second leaf) of SUN(FR−)- and SUN-grown plants was measured at low light intensity. The data display the mean and the bars represent ±s.e.m. calculated from raw data (*n* = 6–7). The *P*-value for the growth treatment was obtained from the mixed model, and Bonferroni-corrected pairwise comparison was conducted between 60FR and 0FR.

At high light intensity, both upper and lower leaves of SUN(FR−)-grown plants had significantly higher leaf net CO_2_ assimilation rates than SUN-grown plants (Fig. 6A, C, respectively). Conversely, at low light intensity, the upper and lower leaves of SUN- and SUN(FR−)-grown plants had similar CO_2_ assimilation rates (Fig. 6B, D, respectively).

With FR light present during measurement (60FR), leaf net CO_2_ assimilation rate (*P*_n,leaf_) was not significantly different in either upper or lower leaves at high light levels [Fig. 6A, C in SUN and SUN(FR−), respectively]. In contrast, at low light intensity, *P*_n,leaf_ was significantly higher with 60FR (Fig. 6B, D). In upper leaves, both SUN- and SUN(FR−)-grown plants increased *P*_n,leaf_ to a similar exent, by 14 and 17 %, respectively (*P*-value for interaction = 0.838; Fig. 6B). In the lower leaves of both SUN- and SUN(FR−)-grown plants, increases of 14 and 12 % in leaf net CO_2_ assimilation rates, respectively, were observed (*P*-value for interaction = 0.885; Fig. 6D).

### The effect of the removal of FR in the actinic light on PSII operating efficiency and NPQ in the upper leaves during transition from high to low light

**Fig. 7.**
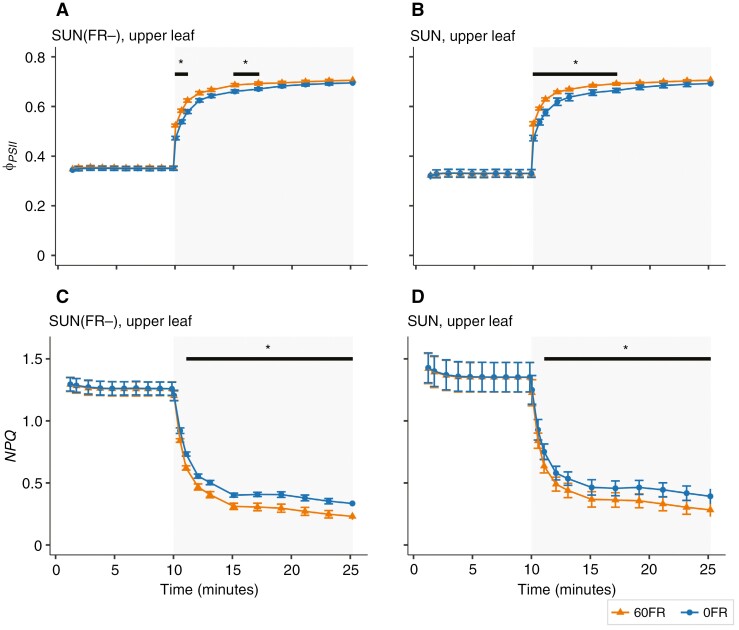
The photosystem II operating efficiency ($\phi_{PSII}$) and non-photochemical quenching (NPQ) response in SUN(FR−) and SUN upper leaves. Leaves were exposed to 10 min of high PPFD at 850 µmol m^−2^ s^−1^ (white area), followed by 15 min at low PPFD at 60 µmol m^−2^ s^−1^ (grey area) in the presence of supplementation of 60 µmol m^−2^ s^−1^ FR (60FR) or absence of FR (0FR) during measurements, as specified in Supplementary Data Table S4. The circles and triangles represent the mean of the data, the solid lines interpolation between the means, and the error bars ±s.e.m. calculated from raw data (*n* = 4–5). *P < 0.05 between 60FR and 0FR (Bonferroni corrected pairwise *t*-test).

To investigate whether removing FR in the actinic light during transition from high to low light influenced the rise in $\phi_{PSII}$ and whether this was associated with a decrease in NPQ, we investigated the chlorophyll fluorescence kinetics during the transition from high to low light intensity (grey area in Fig. 7) in the absence or presence of FR light in the actinic light (60FR and 0FR, respectively, as shown in Fig. 2B; Supplementary Data Table S4). Importantly, unlike gas-exchange measurements, the chlorophyll fluorescence kinetics are not affected by the air mixing time during the transition from high to low light.

In the upper leaves, there was no significant difference in $\phi_{PSII}$ between 60FR and 0FR at the end of the high-light period (*P* = 0.391; Fig. 7A, B) and between SUN(FR−)- or SUN-grown plants (*P* = 0.242). However, during a subsequent 15 min period of low light, 60FR increased $\phi_{PSII}$ for all low light-intensity time points (*P* < 0.05) and this effect was independent of the growth conditions, because it was observed to a similar extent in both SUN and SUN(FR−) upper leaves (*P*-value for interactions > 0.25). However, when examining the impact of 60FR in both SUN and SUN(FR−) separately, 60FR significantly increased $\phi_{PSII}$ only during the first 6 min of low light (Fig. 7A, B).

In a similar manner to $\phi_{PSII}$, the level of NPQ in the upper leaves was similar for 60FR and 0FR (*P* = 0.957) and between SUN and SUN(FR−) plants during the high-light period (*P* = 0.492; Fig. 7C, D). However, during the 15 min of low light, 60FR decreased NPQ compared with 0FR (*P* < 0.05), and the effect was similar between SUN and SUN(FR−) growth conditions (*P*-value for interactions > 0.50). Except for the first two time points, the pairwise comparison in both SUN and SUN(FR−) leaves indicated that 60FR decreased NPQ compared with 0FR (Fig. 7C, D).

### The effect of the presence of FR in the actinic light on PSII operating efficiency and NPQ in the lower leaves during transition from high to low light

**Fig. 8.**
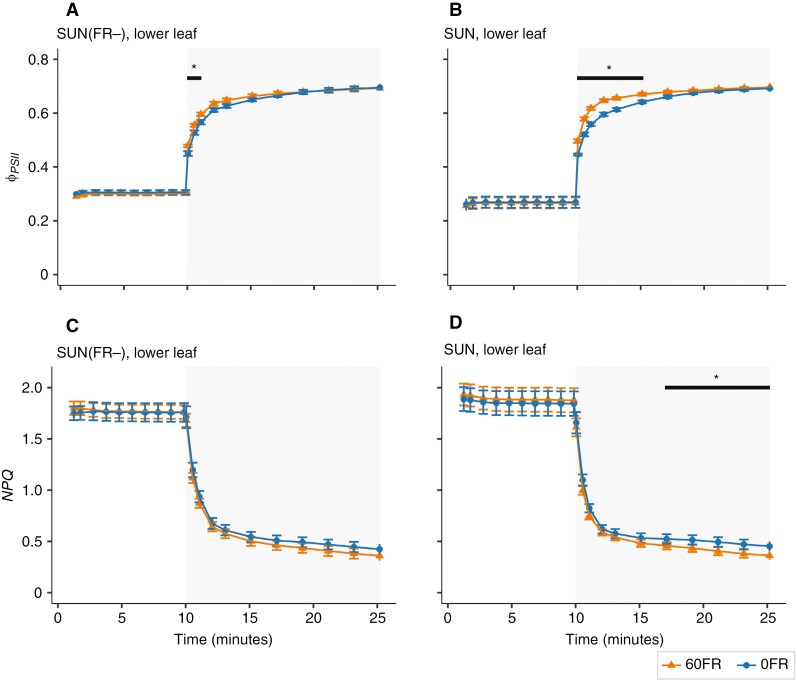
The photosystem II operating efficiency ($\phi_{PSII}$) and non-photochemical quenching (NPQ) response in SUN(FR−) and SUN lower leaves. Leaves were exposed to 10 min of high PPFD at 850 µmol m^−2^ s^−1^, followed by 15 min of low PPFD at 60 µmol m^−2^ s^−1^ in the presence of supplementation of 60 µmol m^−2^ s^−1^ FR (60FR) or absence of FR (0FR) during measurements, as specified in Supplementary Data Table S4. The circles and triangles represent the mean of the data, the solid lines interpolation between the means, and the error bars ±s.e.m. calculated from raw data (*n* = 4–5). *P < 0.05 between 60FR and 0FR (Bonferroni-corrected pairwise *t*-test).

As for the upper leaves, for the lower leaves we analysed the $\phi_{PSII}$ and NPQ during 10 min of high light, followed by 15 min of low light (Fig. 8). During the high-light period, the $\phi_{PSII}$ was similar for 60FR and 0FR (*P* = 0.270) and also between SUN and SUN(FR−) (*P* = 0.146; Fig. 8). However, during a subsequent 15 min period of low light, 60FR increased $\phi_{PSII}$ (*P* < 0.05) for most time points, except for the last four time points (*P* > 0.347). Contrarily to upper leaves, there was an interaction between the long- and short-term effects of FR $\phi_{PSII}$ for the second, third and fourth time points (*P*-value for interactions between 0.0560 and 0.054). There was an increase of $\phi_{PSII}$ in the presence of FR compared with its absence at these time points only in SUN leaves but not in SUN(FR−) leaves. When looking at both SUN and SUN(FR−) separately, 60FR significantly increased $\phi_{PSII}$ in the first 5 min during the transition from high to low light in SUN plants and only in the first 1 min in SUN(FR−)-grown plants (Fig. 8A, B).

The level of NPQ at the end of the high-light period was not significantly different between 60FR and 0FR (*P* = 0.577) or between SUN- and SUN(FR−)-grown plants (*P* = 0.501). During the 15 min of low light, the NPQ was marginally lower with 60FR compared with 0FR for both growth light treatments (Fig. 8C, D). When comparing between 60FR and 0FR in both SUN and SUN(FR−) per time point, 60FR decreased NPQ significantly only during the final 6 min in SUN leaves (Fig. 8D), whereas it did not in SUN(FR−) leaves (Fig. 8C).

### *The increase in* P_*n,leaf*_  *at low light is not always associated with a reduction in NPQ*

**Fig. 9.**
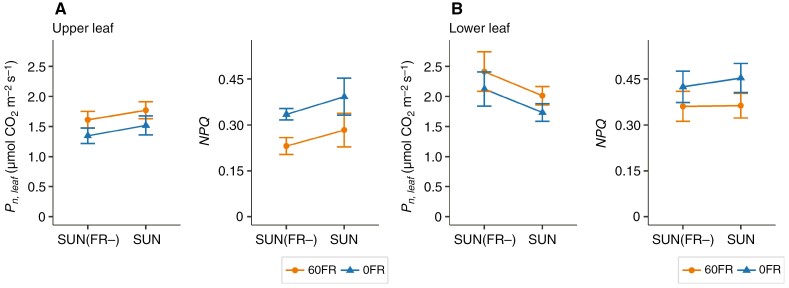
Short- and long-term effects of FR on non-photochemical quenching (NPQ) and net photosynthesis ($P_{n,leaf}$) in upper leaves (A) and lower leaves (B) of young tomato plants grown either with a normal amount (SUN) or with a severely reduced amount [SUN(FR−)] of FR in the solar irradiance. The mean NPQ response and the net CO_2_ assimilation rates ($P_{n,leaf}$, in micromoles of CO_2_ per square metre per second) after 15 min of exposure to low PPFD at 60 µmol m^−2^ s^−1^ in the presence of supplementation of 60 µmol m^−2^ s^−1^ FR (60FR) or absence of FR (0FR) during measurements (Figs 7 and 8) are represented by markers and error bars ±s.e.m. calculated from raw data (*n* = 6–7). Colours indicate measurements made with 60FR and 0FR.

We have shown a reduction in NPQ by 60FR compared with 0FR. This raises the question of whether the enhancement of *P*_n,leaf_ was attributable to a reduction in NPQ. Therefore, we also present the NPQ and *P*_n,leaf_ data simultaneously at one time point at the end of the low light-intensity period (Fig. 9A, B). For both upper and lower leaves, NPQ decreased under 60FR whereas *P*_n,leaf_ increased, displaying a consistent inverse relationship, regardless of whether being grown in SUN or SUN(FR−) conditions (Fig. 9A, B). However, this did not lead to an increase in $\phi_{PSII}$ at the end of the low-light period (Figs 7 and 8).

There was no clear inverse relationship between *P*_n,leaf_ and NPQ across upper and lower leaves when comparing SUN with SUN(FR−) plants. When comparing lower with upper leaves, the lower leaves had higher photosynthetic rates than upper leaves, despite the tendency for higher NPQ in both SUN(FR−) and SUN leaves.

## DISCUSSION

### The impact of the long-term effect of reducing far-red light in the solar irradiance on whole-plant photosynthesis

Plants grown with a reduced amount of FR [SUN(FR−)] were shorter, had smaller and darker green leaves with higher absorptance between 400 and 700 nm (Supplementary Data Fig. S3), and displayed purplish discolorations of leaves and stems in comparison to plants grown under an artificial solar irradiance (SUN; Fig. 3A, B). The increased anthocyanin content is likely to be responsible for the increase in purple coloration. This, in turn, could be a phytochrome-mediated response that is known to regulate the accumulation of anthocyanins in tomato leaves (Liu *et al.*, 2018). Additionally, lower levels of chlorophyll and carotenoid content are frequently observed when plants are grown at low R:FR (Mickens *et al.*, 2018; Kusuma and Bugbee, 2021; Velde and Steppe, 2023). The content of chlorophylls, carotenoids and anthocyanins is responsible for the leaf absorption within the PAR (Pontius *et al.*, 2020). Although pigments were not measured in this study, it is possible that the reduced PAR absorption observed in leaves grown with FR light might result from changes in pigment content.

At high light intensity during measurement, SUN(FR−)- and SUN-grown plants did not differ in whole-plant photosynthetic rates (Fig. 4A). The individual upper and lower leaves of SUN(FR−)-grown plants had significantly higher photosynthetic rates compared with SUN-grown plants at high light intensity (Fig. 6A, C). This observation aligns with a previous study that reported higher photosynthetic rates at high light intensity in tomato leaves grown without FR light in comparison to plants grown with FR light (Wassenaar *et al.*, 2022). The similarity in whole-plant photosynthetic rates can be explained by the higher photosynthetic rates of the individual leaves, which compensated for the reduction in leaf area in SUN(FR−)- compared with SUN-grown plants. Indeed, when normalizing for total leaf area, SUN(FR−)-grown plants exhibited significantly higher whole-plant photosynthetic rates (Fig. 5A).

At low light intensity during measurement, SUN(FR−)-grown plants had lower whole-plant CO_2_ assimilation rates compared with SUN-grown plants (Fig. 4B), despite SUN(FR−)-grown plants being shorter and therefore being, on average, closer to the light source (Supplementary Data Fig. S1). This is consistent with other studies highlighting the effects of FR on morphology by enhancing the ability of plants to capture light through increased leaf area and convert it into biomass at low light intensities (Zhen and Bugbee, 2020*b*; Velde and Steppe, 2023). When normalizing whole-plant CO_2_ assimilation rates by total leaf area, SUN(FR−)- and SUN-grown plants displayed similar rates at low light intensity (Fig. 5B) given the similar leaf CO_2_ assimilation rates at low light (Fig. 6B, D).

It therefore seems that the removal of FR in the long term did not improve whole-plant photosynthesis at high light, despite SUN(FR−)-grown plants having higher leaf photosynthesis per leaf area compared with SUN-grown plants. This result is in line with previous research showing that increasing photosynthetic rates at the leaf level does not necessarily translate into improved whole-plant photosynthesis, as the contribution of individual leaves depends on plant morphology and consequent light interception (Dieleman *et al.*, 2019). To disentangle the contribution of individual leaf layers and stem to whole-plant photosynthesis fully is challenging owing to difficulties related to measurement of light intensity and spectral gradients, including effects of leaf curvature and light scattering from the surrounding surfaces. These estimations are best modelled and require techniques such as ray-tracing simulations (Foo *et al.*, 2020) or modelling light interception and plant morphology (Dieleman *et al.*, 2019), which were beyond the scope of the present study.

### Removal of far-red during measurements decreases leaf photosynthesis but not through reduced photosynthetic efficiency

Reduction of FR in the irradiance during measurement significantly decreased whole-plant CO_2_ assimilation rates at low light intensity compared with the normal amount of FR (Figs 4B and 5B). However, reducing the FR in the irradiance at high light intensities did not reduce whole-plant photosynthesis (Figs 4A and 5A), which is consistent with the measurements of photosynthetic rates at the leaf level (Fig. 6A, C; Supplementary Data Fig. S4). The effect on photosynthesis at low light intensity can be explained by FR photons being directly absorbed and used to drive photosynthesis at limiting light rather than simply the Emerson enhancement effect, because $\phi_{PSII}$ after 10 min of low light intensity was not increased by short-term FR (Figs 7A, B and 8A, B). Likewise, addition of FR (725 nm) also did not affect $\phi_{PSII}$ in lettuce, irrespective of the growth light spectrum (Zhen *et al.*, 2019). In the present study, the effect of directly absorbed FR was expected to be largest at low light intensity, where FR represented ~20 % of the PFD, but less likely at high light intensity because there the short-term application of FR accounted for only a small fraction of the total PFD.

Substituting a fraction of PAR (400–700 nm) with 40 % FR (701–750 nm) was shown to increase whole-plant photosynthesis in several plant species grown at limiting light intensities (Zhen and Bugbee, 2020*a*). However, this effect was shown to be FR wavelength dependent and not supported by tomato leaf photosynthesis measurements above light-limiting light intensities (>500 µmol m^−2^ s^−1^). In contrast, filtering FR (removing 95 % of photons between 701 and 750 nm) from the solar irradiance was shown to reduce leaf corn and sunflower at both high (108.82 % higher than our high light level) and low light intensities (Zhen *et al.*, 2022).

Together, these studies support the enhancement of whole-plant photosynthesis through the substitution of a fraction of PAR with FR. This because absorbed FR photons elicit comparable photosynthetic activity to the PAR photons, as shown by the similar leaf photosynthetic efficiency ($\phi_{CO_{2}}$) (Zhen *et al.*, 2022). In the present study, FR was not substituted, but added to the PAR. Our results show that on an absorbed photon basis between 400 and 800 nm, leaf $\phi_{CO_{2}}$ was negatively affected by adding 60 µmol m^−2^ s^−1^ FR (Supplementary Data Fig. S4). It is worth mentioning that different actinic light spectra were used in the leaf-level measurements and growth treatments. Additionally, the absorption of the FR used during the leaf-level measurements (peak wavelength ~746 nm) was lower than that of the FR of the solar irradiance used during growth and whole-plant photosynthesis measurements (Supplementary Data Tables S6 and S7). Previously, the addition of FR (752 nm) did not enhance $\phi_{PSII}$ in lettuce grown under a red/blue or sunlight spectrum (Zhen *et al.*, 2019). If a similar light spectrum was used in the present study for the leaf-level measurements and for the growth treatment, the increase in leaf photosynthetic CO_2_ assimilation rates when FR is added to the actinic light could, potentially, be larger.

Additionally, the increase in $\phi_{PSII}$ by adding FR at low light intensity during measurements diminished over time (Figs 7A, C and 8A, B). This differed from a previous study, in which short-term FR enhanced $\phi_{PSII}$ throughout the entire low-light phase in *Arabidopsis thaliana* (Kono *et al.*, 2020). This difference could potentially be related to the light spectrum used during measurement, where Kono *et al.* (2020) used red light, with stronger PSII excitation, compared with the white light used in this study (Fig. 2B). Therefore, the difference observed between our study and the study by Kono *et al.* (2020) suggests that the effect of FR on $\phi_{PSII}$ might depend on both the composition of the PAR spectrum and the light intensity used. This potential dependence on the composition of PAR, paired with the previously mentioned results on leaf photosynthetic efficiency, questions the proposition that FR should be included universally in the (extended) PAR.

### The short-term effect of removing far-red light on plant and leaf photosynthesis was independent from growing plants with a reduced or normal amount of far-red in solar irradiance

The lack of interaction between the long-term FR effect and short-term enhancement differs from a previous study investigating the short-term effect of FR on leaves of lettuce grown under sunlight spectrum (using halogen lamps containing FR) and red/blue spectrum (Zhen *et al.*, 2019). In their study, the short-term enhancement with FR at the leaf level during the measurements was greater in red/blue-grown lettuce than in sunlight-grown lettuce, suggesting an interaction effect depending on long-term growth under FR. Our study suggests that this interaction is not universally applicable and depends on the composition of the growth light spectrum.

Interestingly, the kinetics of $\phi_{PSII}$ showed an interaction between short- and long-term FR only in the lower leaves. During the first 5 min of transition from high to low light, 60FR increased $\phi_{PSII}$ more in the lower leaves of SUN-grown plants than in SUN(FR−)-grown plants (Fig. 8A, B). In contrast, in the upper leaves, this effect was similar in both SUN and SUN(FR−) leaves (Fig. 7A, B).

It has been shown that when shade leaves were illuminated with FR light, they had a higher $\phi_{PSII}$ than the same leaves illuminated in the absence of FR light (Taylor *et al.*, 2019). It has also been reported that the growth light spectrum has the potential to influence the $\phi_{PSII}$ as a result of changes in PSI and PSII stoichiometry. For example, shade-grown leaves of cucumber illuminated with FR had higher $\phi_{PSII}$ than sun-grown leaves and a relatively greater number of PSII reaction centres (Hogewoning *et al.*, 2012). Although not within the scope of the present study, our observed differences in $\phi_{PSII}$ could, potentially, be explained by changes in the photosystem stoichiometry, potentially arising from the combination of long-term effects of low light and low PSS [PSS = 0.66 vs. PSS = 0.81, in SUN and SUN(FR−) lower leaves, respectively], which might become larger in older plants with stronger gradients in light intensity and spectrum.

### Limitations in leaf CO_2_ assimilation rates at low light are explained only in part by NPQ

Removal of FR during the measurements increased NPQ in both the upper and lower leaves at the end of the low-light period (Figs 7C, D and 8C, D). However, the lower leaves in SUN(FR−)-grown plants, despite the higher NPQ levels, exhibited higher leaf CO_2_ assimilation rates than SUN-grown leaves at low light intensity (Figs 7C, D, 8C, D and 9). The higher leaf CO_2_ assimilation in lower leaves compared with upper leaves despite the higher NPQ could largely be explained by the lower *R*_d_ (Supplementary Data Fig. S5). Additionally, ${F^{\prime }}_{q}/{F^{\prime }}_{v}\;$ and $\phi_{PSII}$ in the presence of 60FR reached a steady state more quickly than in the absence of 0FR, while NPQ was still decreasing (Figs 7A, B and 8A, B; Supplementary Data Fig. S6). Although not measured in this study, the reduction in NPQ by 60FR could be explained by its effect on the ΔpH gradient through the oxidation of the plastoquinone pool. FR preferentially excites PSI, which promotes plastoquinone and Q_A_ oxidation, and lowers the ΔpH (Baker, 2008; Kono *et al.*, 2020). In turn, this deactivates PsbS protonation and favours violaxanthin epoxidation, resulting in a decrease in NPQ and an increase in $\phi_{PSII}$, as observed at first in response to 60FR when lowering light intensity. However, contrary to expectation, NPQ remained decreased, but $\phi_{PSII}$ was no longer increased in response to 60FR at the end of the low-light period (Figs 7 and 8). Therefore, our results suggest that the $\phi_{PSII}$ at the end of the low-light period is not limited primarily by NPQ, but rather constrained by the ability of the photosynthetic apparatus to drive photosynthesis (Baker, 2008; Yamori, 2016). This finding suggests some divergence from the notion that NPQ limits, to a large extent, the recovery of photosynthetic rates during the transition from high to low light intensity (Kromdijk *et al.*, 2016; De Souza *et al.*, 2022). In turn, this is important for scaling individual leaf responses to fluctuating lights from leaf to whole-plant photosynthesis.

## Conclusions

This study is the first to quantify the impact of short-term removal of FR in plants grown with a severely reduced amount of FR in the solar irradiance compared with plants grown with normal amounts of FR. Plants grown with severely reduced far-red display remarkably lower whole-plant CO_2_ assimilation rates at low light intensities in comparison to plants grown with a normal FR level in the irradiance. However, at high light intensities, these plants performed in a similar way to plants grown with a normal amount of FR. This observation was associated with higher leaf CO_2_ assimilation rates at high light intensity, and therefore challenges the idea that FR in solar irradiance improves whole-plant photosynthesis through a more efficient use of FR for leaf photosynthesis. Furthermore, the removal of FR did not reduce leaf CO_2_ assimilation rates through a reduction in $\phi_{PSII}$ at the end of a low light-intensity period following a short period of high light, regardless of the leaf position and growth light treatment. These findings suggest that in young plants, the presence of FR in the solar irradiance increases whole-plant photosynthesis in tomato, but only at low light intensity. In the long term, however, factors such as leaf area might have a more important role than the short-term enhancement of photosynthesis by the presence of FR light.

## SUPPLEMENTARY DATA

Supplementary data are available at *Annals of Botany* online and consist of the following.

Figure S1: vertical light-intensity gradient of the photosynthetic photon flux (PPFD, 400–700 nm) measured inside the chamber used to measure whole-plant CO_2_ assimilation rates without a plant present. The high PPFD (A) and low PPFD (B) were set at the top of the plant to 660 and 100 µmol m^-2^ s^-1^, respectively. Lines display the mean and the ribbon the SEM (n = 6), according to a quadratic regression fitted to measurements of the vertical light profile. Figure S2: horizontal light-intensity gradient of the photosynthetic photon flux (PPFD, 400–700 nm) measured inside the chamber used for measurement of whole-plant CO_2_ assimilation rates. The circle represents a top view of the chamber with the plant centered and high PPFD set to 660 µmol m^-2^ s^-1^ at the top of the plant for high light. Figure S3: spectral absorptance of upper (A) and lower (B) leaves from SUN- and SUN(FR−)-grown plants (*n* = 6); s.e.m. is smaller than the thickness of the line. The mean absorptance between 400-700 nm for lower leaves was 0.882 and 0.921 for SUN and SUN(FR-), respectively. For upper leaves 0.889 and 0.917 for SUN and SUN(FR-), respectively. See Table S6 for the resulting difference in fraction absorbed light for the different light spectra used. Figure S4: quantum yield of CO_2_ assimilation ($\phi_{CO_{2\;\;\;}}$, in µmol gross CO_2_ µmol absorbed photons^-1^) based on absorbed photons in the range 400–700 nm in A and B; and based on absorbed photons in the range 400–800 nm in C and D. Symbols represent the mean and error bars represent the SEM (n = 6-7). The PPFD was 60 µmol m^-2^ s^-1^, and the FR (700-800 nm) was 60 µmol m^-2^ s^-1^. Figure S5: dark respiration rates (*R*_d_) of lower and upper leaves of SUN(FR−)- and SUN-grown plants (*n* = 6). Panel A. Linear relationship between leaf area and dark respiration, represented by the solid line with a 95% confidence interval represented by the grey area, panel B. Colour indicates the growth light treatment. Circles and squares represent upper and lower leaves, respectively. The *p-value* was obtained by a t-test for the correlation coefficient. Figure S6: ${F^{\prime }}_{q}/{F^{\prime }}_{v}\;$ response during 10 min high PPFD at 850 µmol m^−2^ s^−1^ (white area) and 15 min low PPFD at 60 µmol m^−2^ s^−1^ (grey area) in upper and lower leaves of SUN- and SUN(FR−)-grown plants. Colours indicate the presence of supplementation of 60 µmol m^-2^ s^-1^ or absence of FR light (60FR and 0FR) during the measurements. Upper leaf layer, SUN(FR-) grown in panel A, upper leaf layer, SUN grown in panel B, lower leaf layer, SUN(FR-) grown in panel C, lower leaf layer, SUN grown in panel D. Arrows indicate time points at which statistical analysis was done. The symbols represent the mean of the data, the solid line interpolation between the means, and the error bar ± SEM calculated from raw data (n = 4-5). Table S1: light intensity and spectrum during growth corresponding to the spectrum shown in Fig. 1B. The R:FR ratio was calculated considering the ranges between 660 – 670 nm for R light, and 725 – 735 nm for FR. Table S2: artificial solar irradiance (Fig. 2A) used during measurement for whole-plant CO_2_ assimilation rates under high and low light intensity. The R:FR ratio was calculated considering the ranges between 660 – 670 nm for R light, and 725 – 735 nm for FR. Table S3: chamber environmental conditions of the sample cell containing the plant during whole-plant gas exchange-measurements at high and low light intensity. Table S4: measuring light irradiance (Fig. 2B) used for chlorophyll fluorescence and leaf CO_2_ assimilation rates under high to low light intensity. Table S5: chamber environmental conditions in the cuvette of the LI-6400 portable gas-exchange system used during leaf-level gas exchange at high and low light intensity; given that there was little difference between FR+ and FR−, they are averaged here for simplicity. Table S6: fraction of absorbed light under the artificial solar spectrum of the upper and lower leaves of SUN- and SUN(FR−)-grown plants with a normal intensity of far-red light (FR+) and a severely reduced intensity (FR−). Table S7: fraction of absorbed light under the white light actinic spectrum of the upper and lower leaves of SUN- and SUN(FR−)-grown plants at the low light intensity used during combined leaf gas-exchange and chlorophyll-fluorescence measurements with 0FR and 60FR.

mcae104_suppl_Supplementary_Figure_S1

mcae104_suppl_Supplementary_Figure_S2

mcae104_suppl_Supplementary_Figure_S3

mcae104_suppl_Supplementary_Figure_S4

mcae104_suppl_Supplementary_Figure_S5

mcae104_suppl_Supplementary_Figure_S6

mcae104_suppl_Supplementary_Materials

## References

[CIT0001] Baker NR. 2008. Chlorophyll fluorescence: a probe of photosynthesis in vivo. Annual Review of Plant Biology 59: 89–113. 10.1146/annurev.arplant.59.032607.092759 18444897

[CIT0002] Bates D, Mächler M, Bolker BM, Walker SC. 2015. Fitting linear mixed-effects models using lme4. Journal of Statistical Software 67: 1–48.

[CIT0003] Bou-Torrent J, Toledo-Ortiz G, Ortiz-Alcaide M, et al 2015. Regulation of carotenoid biosynthesis by shade relies on specific subsets of antagonistic transcription factors and cofactors. Plant Physiology 169: 1584–1594. 26082398 10.1104/pp.15.00552 PMC4634050

[CIT0004] Chow WS, Melis A, Anderson JM. 1990. Adjustments of photosystem stoichiometry in chloroplasts improve the quantum efficiency of photosynthesis. Proceedings of the National Academy of Sciences of the United States of America 87: 7502–7506. 11607105 10.1073/pnas.87.19.7502 PMC54775

[CIT0005] Demotes-Mainard S, Péron T, Corot A, et al 2016. Plant responses to red and far-red lights, applications in horticulture. Environmental and Experimental Botany 121: 4–21.

[CIT0029] De Souza AP, Burgess SJ, Doran L, et al 2022. Soybean photosynthesis and crop yield are improved by accelerating recovery from photoprotection. Science 377: 851–854. 35981033 10.1126/science.adc9831

[CIT0006] Dieleman JA, De Visser PHB, Meinen E, Grit JG, Dueck TA. 2019. Integrating morphological and physiological responses of tomato plants to light quality to the crop level by 3D modeling. Frontiers in Plant Science 10: 839. 31354751 10.3389/fpls.2019.00839 PMC6637845

[CIT0007] Emerson R, Chalmers R, Cederstrand C. 1957. Some factors influencing the long-wave limit of photosynthesis. Proceedings of the National Academy of Sciences of the United States of America 43: 133–143. 16589986 10.1073/pnas.43.1.133 PMC528397

[CIT0008] Evans JR. 1986. A quantitative analysis of light distribution between the two photosystems, considering variation in both the relative amounts of the chlorophyll–protein complexes and the spectral quality of light. Photobiochemistry and Photobiophysics 10: 135–147.

[CIT0009] Evans JR. 1987. The dependence of quantum yield on wavelength and growth irradiance. Functional Plant Biology 14: 69.

[CIT0010] Field CB, Ball JT, Berry JA. 1989. Measurement of transpiration and leaf conductance. In: Pearcy RW et al eds. Plant physiological ecology: field methods and instrumentation. Dordrecht: Springer, 209–253.

[CIT0011] Foo CC, Burgess AJ, Retkute R, Tree-Intong P, Ruban AV, Murchie EH. 2020. Photoprotective energy dissipation is greater in the lower, not the upper, regions of a rice canopy: a 3D analysis. Journal of Experimental Botany 71: 7382–7392. 32905587 10.1093/jxb/eraa411 PMC7906788

[CIT0012] Hogewoning SW, Douwstra P, Trouwborst G, Van Ieperen W, Harbinson J. 2010. An artificial solar spectrum substantially alters plant development compared with usual climate room irradiance spectra. Journal of Experimental Botany 61: 1267–1276. 20202994 10.1093/jxb/erq005

[CIT0013] Hogewoning SW, Wientjes E, Douwstra P, et al 2012. Photosynthetic quantum yield dynamics: from photosystems to leaves. Plant Cell 24: 1921–1935. 22623496 10.1105/tpc.112.097972 PMC3442578

[CIT0014] Hu C, Nawrocki WJ, Croce R. 2021. Long-term adaptation of *Arabidopsis thaliana* to far-red light. Plant Cell and Environment 44: 3002–3014. 10.1111/pce.14032 PMC8453498 33599977

[CIT0015] Ji Y, Ouzounis T, Courbier S, et al 2019. Far-red radiation increases dry mass partitioning to fruits but reduces *Botrytis cinerea* resistance in tomato. Environmental and Experimental Botany 168: 103889.

[CIT0016] Kalaitzoglou P, van Ieperen W, Harbinson J, et al 2019. Effects of continuous or end-of-day far-red light on tomato plant growth, morphology, light absorption, and fruit production. Frontiers in Plant Science 10: 322. 30984211 10.3389/fpls.2019.00322 PMC6448094

[CIT0017] Kono M, Kawaguchi H, Mizusawa N, Yamori W, Suzuki Y, Terashima I. 2020. Far-red light accelerates photosynthesis in the low-light phases of fluctuating light. Plant and Cell Physiology 61: 192–202. 31617558 10.1093/pcp/pcz191

[CIT0018] Kromdijk J, Głowacka K, Leonelli L, et al 2016. Improving photosynthesis and crop productivity by accelerating recovery from photoprotection. Science 354: 857–861. 27856901 10.1126/science.aai8878

[CIT0019] Kusuma P, Bugbee B. 2021. Far-red fraction: an improved metric for characterizing phytochrome effects on morphology. Journal of the American Society for Horticultural Science 146: 3–13.

[CIT0020] Kuznetsova A, Brockhoff PB, Christensen RHB. 2017. lmerTest Package: tests in linear mixed effects models. Journal of Statistical Software 82: 1–26.

[CIT0021] Lenth RV. 2016. Least-squares means: the R package lsmeans. Journal of Statistical Software 69: 1–33.

[CIT0022] Liu CC, Chi C, Jin LJ, Zhu J, Yu JQ, Zhou YH. 2018. The bZip transcription factor *HY5* mediates *CRY1a*-induced anthocyanin biosynthesis in tomato. Plant Cell and Environment 41: 1762–1775. 10.1111/pce.13171 29566255

[CIT0023] McCree KJ. 1971. The action spectrum, absorptance and quantum yield of photosynthesis in crop plants. Agricultural Meteorology 9: 191–216.

[CIT0024] Mickens MA, Skoog EJ, Reese LE, et al 2018. A strategic approach for investigating light recipes for ‘Outredgeous’ red romaine lettuce using white and monochromatic LEDs. Life Sciences in Space Research 19: 53–62. 30482283 10.1016/j.lssr.2018.09.003

[CIT0025] Oxborough K, Baker NR. 1997. Resolving chlorophyll a fluorescence images of photosynthetic efficiency into photochemical and non-photochemical components – calculation of qP and Fv-/Fm-; without measuring Fo. Photosynthesis Research 54: 135–142.

[CIT0026] Pontius J, Schaberg P, Hanavan R. 2020. Remote sensing for early, detailed, and accurate detection of forest disturbance and decline for protection of biodiversity. In: Cavender-Bares J, Gamon JA, Townsend PA. eds. Remote sensing of plant biodiversity. Cham, Switzerland: Springer, 121–154.

[CIT0027] Sager JC, Smith WO, Edwards JL, Cyr KL. 1988. Photosynthetic efficiency and phytochrome photoequilibria determination using spectral data. Transactions of the ASAE 31: 1882–1889.

[CIT0028] Schneider CA, Rasband WS, Eliceiri KW. 2012. NIH Image to ImageJ: 25 years of image analysis. Nature Methods 9: 671–675. 22930834 10.1038/nmeth.2089 PMC5554542

[CIT0030] Taylor CR, Van Ieperen W, Harbinson J. 2019. Demonstration of a relationship between state transitions and photosynthetic efficiency in a higher plant. The Biochemical Journal 476: 3295–3312. 31694051 10.1042/BCJ20190576 PMC6854431

[CIT0031] Trouwborst G, Hogewoning SW, Harbinson J, van Ieperen W. 2011. The influence of light intensity and leaf age on the photosynthetic capacity of leaves within a tomato canopy. Journal of Horticultural Science and Biotechnology 86: 403–407.

[CIT0032] Velde EVD, Steppe K. 2023. Leaf morphology, optical characteristics and phytochemical traits of butterhead lettuce affected by increasing the far-red photon flux. Frontiers in Plant Science 14: 1129335. 37600174 10.3389/fpls.2023.1129335 PMC10433762

[CIT0033] Wassenaar MLJ, van Ieperen W, Driever SM. 2022. Low red to far-red ratio increases resistance to CO_2_ diffusion and reduces photosynthetic efficiency in low light grown tomato plants. Environmental and Experimental Botany 200: 104918.

[CIT0034] Wickham H, Averick M, Bryan J, et al 2019. Welcome to the Tidyverse. Journal of Open Source Software 4: 1686.

[CIT0035] Yamori W. 2016. Photosynthetic response to fluctuating environments and photoprotective strategies under abiotic stress. Journal of Plant Research 129: 379–395. 27023791 10.1007/s10265-016-0816-1

[CIT0036] Zhang N, Van Westreenen A, Anten NPR, Evers JB, Marcelis LFM. 2020. Disentangling the effects of photosynthetically active radiation and red to far-red ratio on plant photosynthesis under canopy shading: a simulation study using a functional–structural plant model. Annals of Botany 126: 635–646. 31793625 10.1093/aob/mcz197 PMC7489061

[CIT0037] Zhen S, Bugbee B. 2020a. Far-red photons have equivalent efficiency to traditional photosynthetic photons: implications for redefining photosynthetically active radiation. Plant Cell and Environment 43: 1259–1272. 10.1111/pce.13730 31990071

[CIT0038] Zhen S, Bugbee B. 2020b. Substituting far-red for traditionally defined photosynthetic photons results in equal canopy quantum yield for CO_2_ fixation and increased photon capture during long-term studies: implications for re-defining PAR. Frontiers in Plant Science 11: 581156. 33014004 10.3389/fpls.2020.581156 PMC7516038

[CIT0040] Zhen S, van Iersel MW. 2017. Far-red light is needed for efficient photochemistry and photosynthesis. Journal of Plant Physiology 209: 115–122. 28039776 10.1016/j.jplph.2016.12.004

[CIT0039] Zhen S, Haidekker M, van Iersel MW. 2019. Far-red light enhances photochemical efficiency in a wavelength-dependent manner. Physiologia Plantarum 167: 21–33. 30203475 10.1111/ppl.12834

[CIT0041] Zhen S, van Iersel MW, Bugbee B. 2022. Photosynthesis in sun and shade: the surprising importance of far-red photons. The New Phytologist 236: 538–546. 35832002 10.1111/nph.18375

