# Supplementary figures and images for: Far-red light effects on plant photosynthesis: from short-term enhancements to long-term effects of artificial solar light
Source: Ann Bot. 2024 Jul 1;135(3):589–602. doi: 10.1093/aob/mcae104 (PMC11897601; doi:10.1093/aob/mcae104)

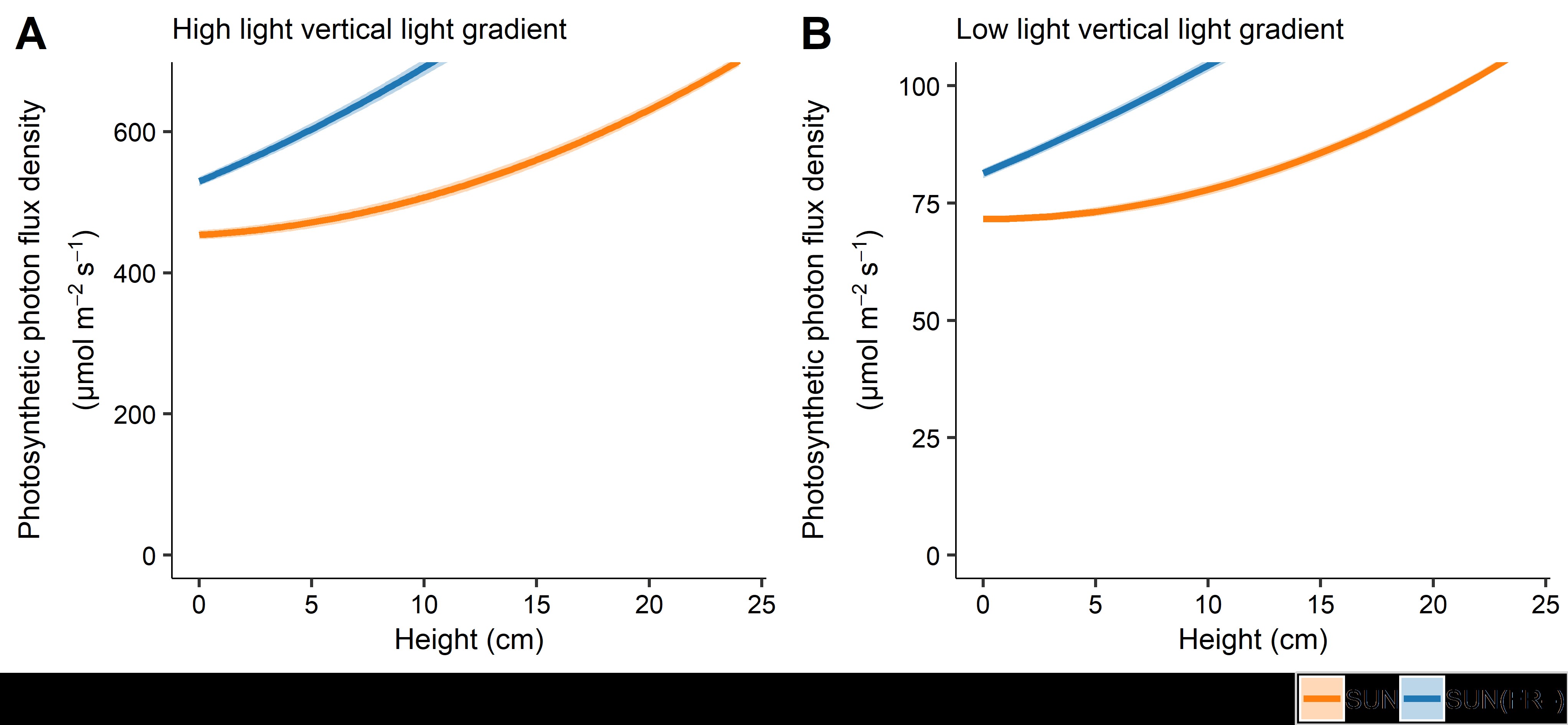

Supplement: mcae104_suppl_Supplementary_Figure_S1 [file mcae104_suppl_supplementary_figure_s1.jpeg]

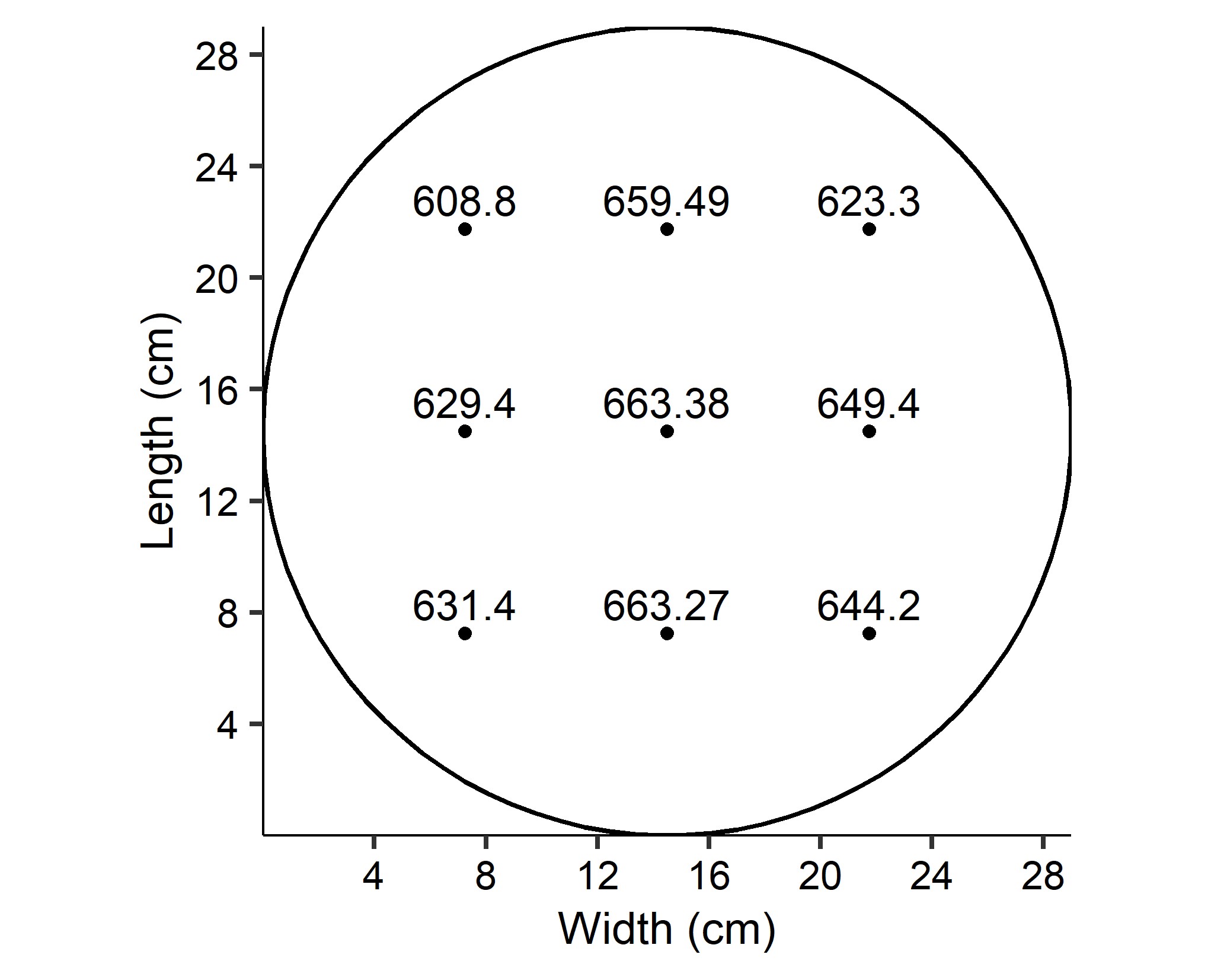

Supplement: mcae104_suppl_Supplementary_Figure_S2 [file mcae104_suppl_supplementary_figure_s2.jpeg]

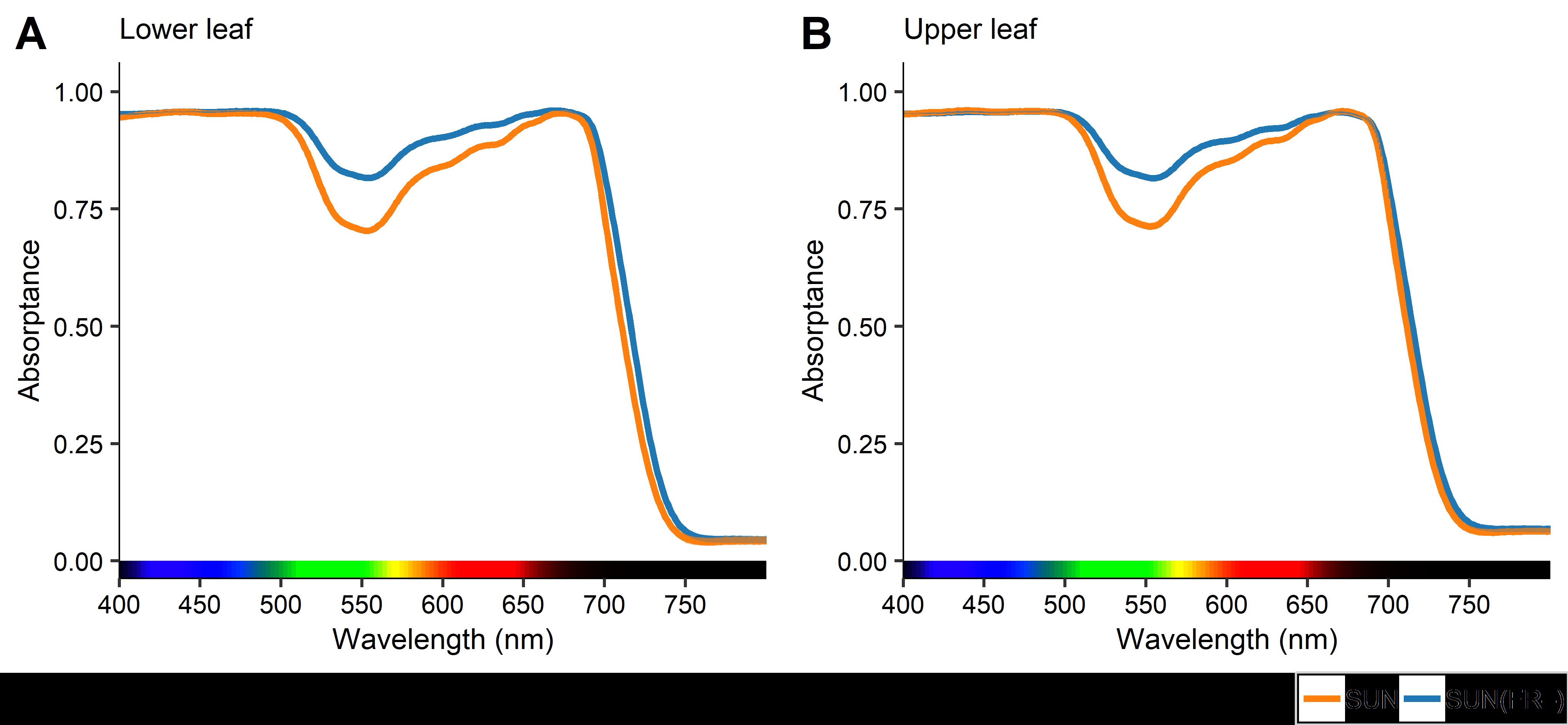

Supplement: mcae104_suppl_Supplementary_Figure_S3 [file mcae104_suppl_supplementary_figure_s3.jpeg]

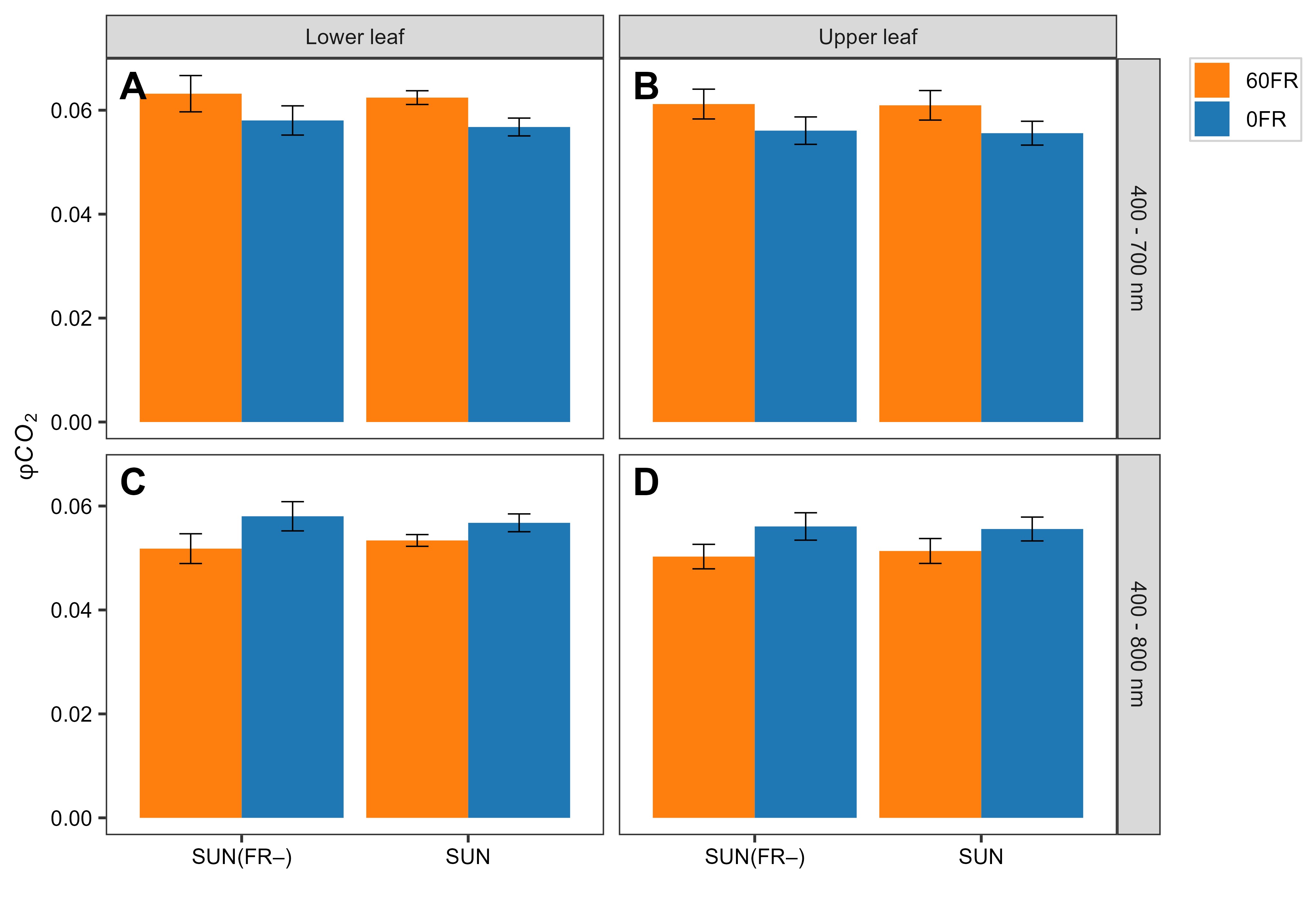

Supplement: mcae104_suppl_Supplementary_Figure_S4 [file mcae104_suppl_supplementary_figure_s4.jpeg]

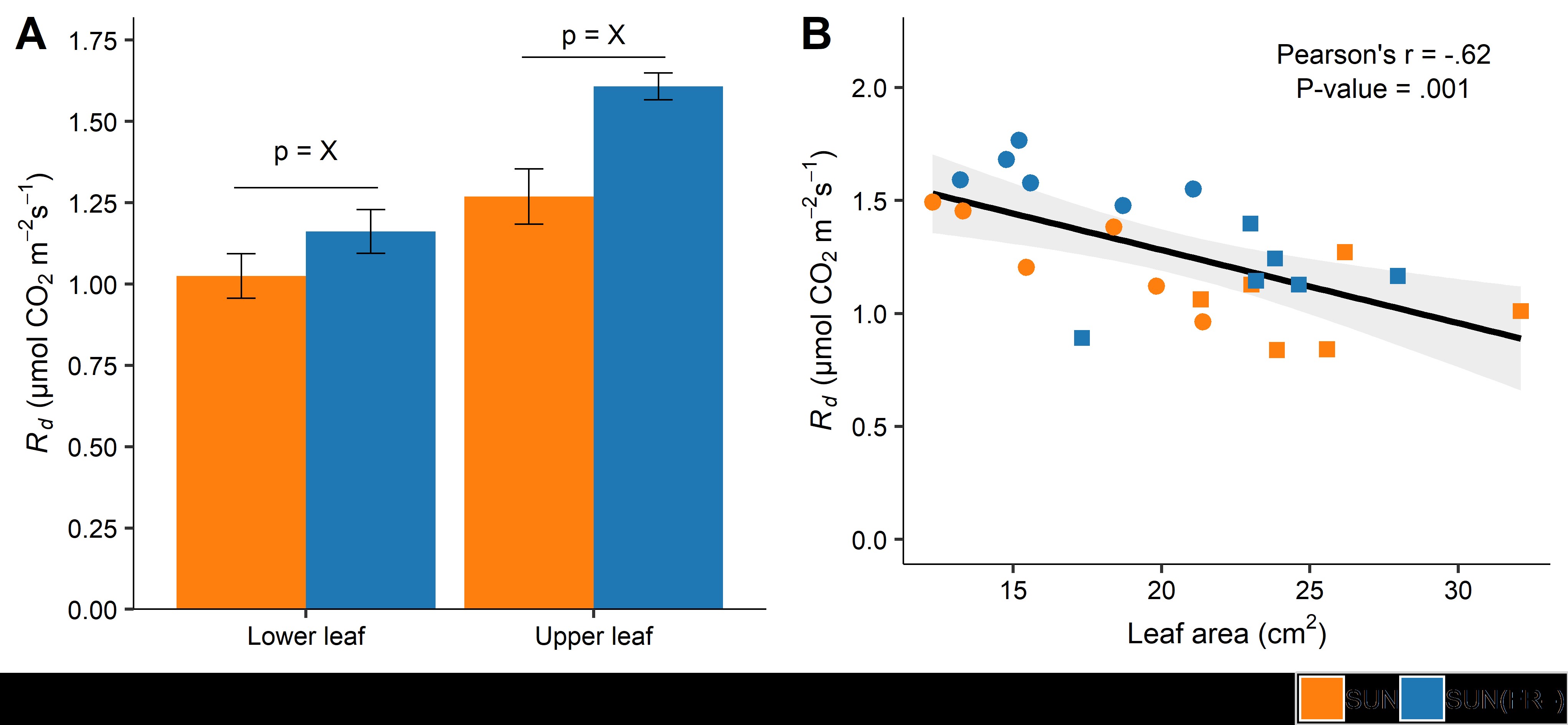

Supplement: mcae104_suppl_Supplementary_Figure_S5 [file mcae104_suppl_supplementary_figure_s5.jpeg]

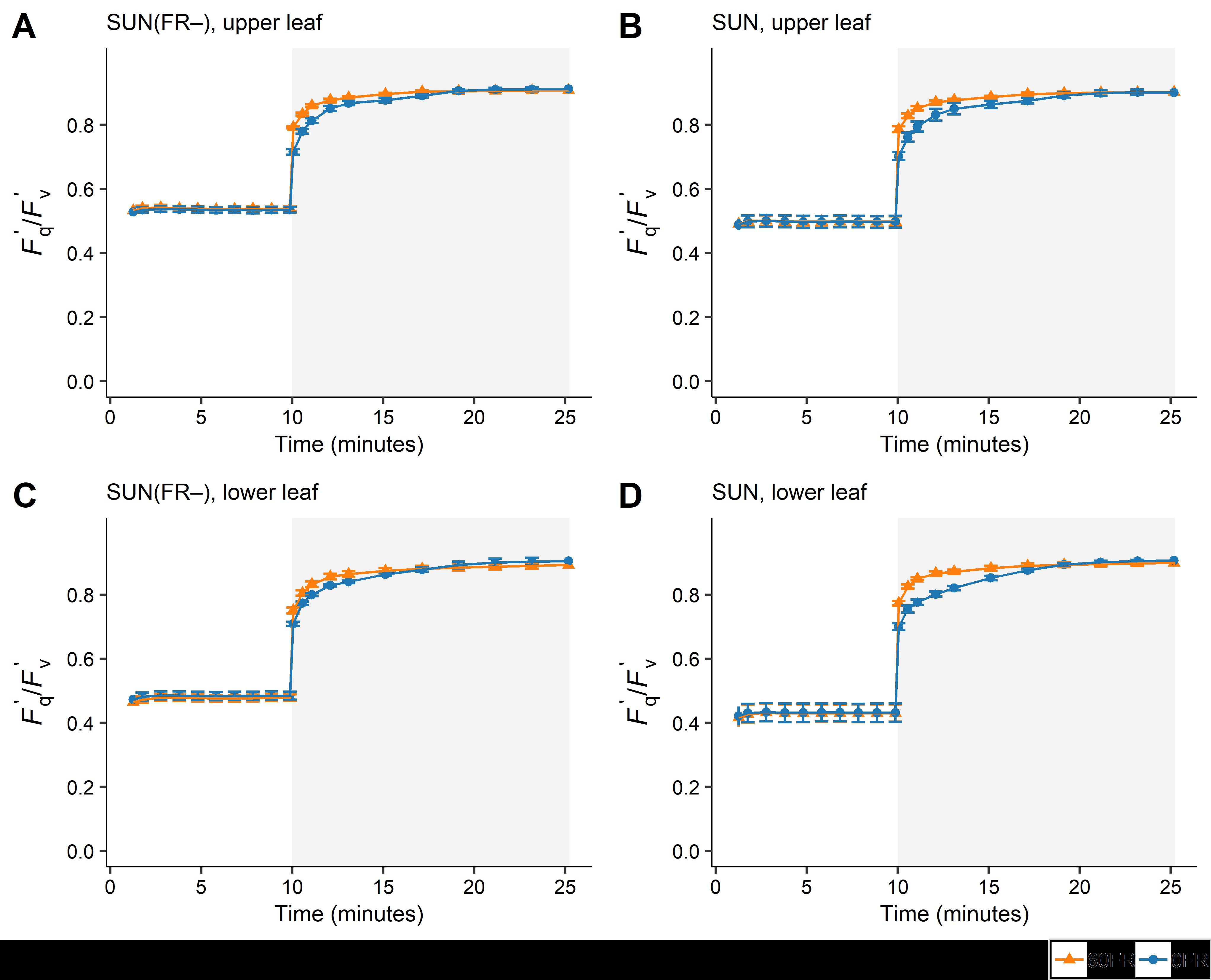

Supplement: mcae104_suppl_Supplementary_Figure_S6 [file mcae104_suppl_supplementary_figure_s6.jpeg]
