# Supplementary material for: Far-red light effects on plant photosynthesis: from short-term enhancements to long-term effects of artificial solar light
Source: Ann Bot. 2024 Jul 1;135(3):589–602. doi: 10.1093/aob/mcae104 (PMC11897601; doi:10.1093/aob/mcae104)
Supplement: mcae104_suppl_Supplementary_Materials [file mcae104_suppl_supplementary_materials.docx]

**Table S1.** Light intensity and spectrum during growth corresponding to the spectrum shown in Figure 1B. The R:FR ratio was calculated considering the ranges between 660 – 670 nm for R light, and 725 – 735 nm for FR.

| **Parameter** | **Unit** | **Growth light treatment** | |
| --- | --- | --- | --- |
|  |  | **SUN** | **SUN(FR-)** |
| Maximum PPFD | µmol m^-2^ s^-1^ | 325 | 325 |
| PFD (400-750) | µmol m^-2^ s^-1^ | 386 | 336 |
| FR (700-750) | µmol m^-2^ s^-1^ | 61 | 12 |
| PSS |  | 0.72 | 0.83 |
| R:FR |  | 1.1 | 6.8 |

**Table S2.** Artificial solar irradiance (Figure 2A) used during measurement for whole-plant CO_2_ assimilation rates under high and low light intensity. The R:FR ratio was calculated considering the ranges between 660 – 670 nm for R light, and 725 – 735 nm for FR.

| **Label of artificial solar light source** | **PPFD**  **(400-700 nm) µmol m^-2^ s^-1^** | **PFD**  **(400-750 nm) µmol m^-2^ s^-1^** | **FR**  **(700-750 nm) µmol m^-2^ s^-1^** | **PSS and R:FR** | **Light intensity** |
| --- | --- | --- | --- | --- | --- |
| **FR^-^** | 660 | 683 | 24 | PSS=0.83  R:FR=6.8 | High light |
| **FR^-^** | 100 | 104 | 4 | PSS=0.83  R:FR=6.8 | Low light |
| **FR^+^** | 660 | 783 | 124 | PSS=0.72  R:FR=1.1 | High light |
| **FR^+^** | 100 | 119 | 19 | PSS=0.72  R:FR=1.1 | Low light |

**Table S3.** Measuring light irradiance (Figure 2B) used for chlorophyll fluorescence and leaf CO_2_ assimilation rates under high to low light intensity

| **Label of actinic light source** | **PPFD**  **(400-700 nm) µmol m^-2^ s^-1^** | **PFD**  **(400-750 nm) µmol m^-2^ s^-1^** | **FR**  **(700-750 nm) µmol m^-2^ s^-1^** | **PSS and R:FR** | **Light intensity** |
| --- | --- | --- | --- | --- | --- |
| **0FR** | 850 | 850 | 0 | PSS=0.85  R:FR=2.4 | High light |
| **0FR** | 60 | 60 | 0 | PSS=0.85  R:FR=2.4 | Low light |
| **60FR** | 850 | 910 | 60 | PSS=0.76  R:FR=0.014 | High light |
| **60FR** | 60 | 119 | 60 | PSS=0.35  R:FR=0.004 | Low light |

**Table S4.** Chamber environmental conditions of the sample cell containing the plant during whole-plant gas exchange measurements at high and low light intensity.

| **Measurement spectrum** | **Growth spectrum** | **Light phase** | **CO_2_ sample cell**  **(µmol mol^-1^)** | **Relative humidity (%)** | **Temperature (°C)** |
| --- | --- | --- | --- | --- | --- |
| FR+ | SUN(FR-) | High light | 374.36 ± 5.55 | 72 ± 4 | 27.02 ± 0.77 |
| FR+ | SUN(FR-) | Low light | 381.83 ± 1.6 | 68 ± 5 | 25.31 ± 0.7 |
| FR+ | SUN | High light | 369.23 ± 8.51 | 73 ± 2 | 26.44 ± 0.2 |
| FR+ | SUN | Low light | 376.26 ± 2.88 | 69 ± 2 | 24.93 ± 0.19 |
| FR- | SUN(FR-) | High light | 375.46 ± 4.84 | 73 ± 4 | 26.58 ± 0.67 |
| FR- | SUN(FR-) | Low light | 383.7 ± 1.35 | 68 ± 3 | 25.13 ± 0.59 |
| FR- | SUN | High light | 370.23 ± 8.35 | 73 ± 2 | 26.44 ± 0.49 |
| FR- | SUN | Low light | 378.39 ± 2.38 | 68 ± 2 | 25.03 ± 0.33 |

**Table S5.** Chamber environmental conditions in the cuvette of the LI-6400 portable gas exchange system used during leaf-level gas exchange at high and low light intensity, as there was little difference between FR+ and FR- they are averaged here for simplicity.

| **Leaf layer** | **Growth spectrum** | **Light phase** | **CO_2_ sample cell**  **(µmol mol^-1^)** | **Relative humidity (%)** | **Leaf temperature (°C)** |
| --- | --- | --- | --- | --- | --- |
| Lower leaf | SUN(FR-) | High light | 364 ± 2.00 | 75 ± 4.89 | 23.30 ± 0.76 |
| Lower leaf | SUN(FR-) | Low light | 395 ± 2.47 | 62 ± 9.82 | 22.73 ± 0.51 |
| Lower leaf | SUN | High light | 372 ± 4.48 | 73 ± 3.07 | 23.82 ± 0.81 |
| Lower leaf | SUN | Low light | 396 ± 1.59 | 62 ± 6.33 | 22.92 ± 0.51 |
| Upper leaf | SUN(FR-) | High light | 362 ± 1.08 | 72 ± 1.90 | 23.07 ± 0.79 |
| Upper leaf | SUN(FR-) | Low light | 397 ± 1.73 | 56 ± 4.54 | 22.69 ± 0.51 |
| Upper leaf | SUN | High light | 365 ± 2.73 | 72 ± 1.96 | 23.43 ± 0.76 |
| Upper leaf | SUN | Low light | 396 ± 1.90 | 55 ± 5.69 | 22.84 ± 0.58 |

**Table S6.** Fraction absorbed light under the artificial solar spectrum of the upper and lower leaves of SUN and SUN(FR-) grown plants with a normal intensity of far-red light (FR+) and a severely reduced intensity (FR-).

|  | **FR+**  (measurement spectrum) | **FR-**  (measurement spectrum) |
| --- | --- | --- |
| **SUN grown**  **Lower leaves** |  |  |
| 400-700 nm | 0.876 | 0.876 |
| 400-800 nm | 0.730 | 0.850 |
| 701-800 nm | 0.201 | 0.286 |
|  |  |  |
| **SUN(FR-) grown**  **Lower leaves** |  |  |
| 400-700 nm | 0.918 | 0.918 |
| 400-800 nm | 0.774 | 0.893 |
| 701-800 nm | 0.253 | 0.335 |
|  |  |  |
| **SUN grown**  **Upper leaves** |  |  |
| 400-700 nm | 0.884 | 0.884 |
| 400-800 nm | 0.740 | 0.858 |
| 701-800 nm | 0.217 | 0.300 |
|  |  |  |
| **SUN(FR-) grown**  **Upper leaves** |  |  |
| 400-700 nm | 0.914 | 0.915 |
| 400-800 nm | 0.771 | 0.890 |
| 701-800 nm | 0.253 | 0.346 |

**Table S7.** Fraction absorbed light under the white light actinic spectrum of the upper and lower leaves of SUN and SUN(FR-) grown plants at low light intensity used during combined leaf gas-exchange and chlorophyll fluorescence measurements with 0FR and 60FR.

|  | **60FR**  (measurement spectrum) | **0FR**  (measurement spectrum) |
| --- | --- | --- |
| **SUN grown**  **Lower leaves** |  |  |
| 400-700 nm | 0.839 | 0.838 |
| 400-800 nm | 0.490 | 0.838 |
| 701-800 nm | 0.161 | / |
|  |  |  |
| **SUN(FR-) grown**  **Lower leaves** |  |  |
| 400-700 nm | 0.897 | 0.897 |
| 400-800 nm | 0.547 | 0.897 |
| 701-800 nm | 0.216 | / |
|  |  |  |
| **SUN grown**  **Upper leaves** |  |  |
| 400-700 nm | 0.847 | 0.846 |
| 400-800 nm | 0.502 | 0.846 |
| 701-800 nm | 0.177 | / |
|  |  |  |
| **SUN(FR-) grown**  **Upper leaves** |  |  |
| 400-700 nm | 0.894 | 0.894 |
| 400-800 nm | 0.544 | 0.894 |
| 701-800 nm | 0.215 | / |
